# Improving the production of carbamoyltobramycin by an industrial *Streptoalloteichus tenebrarius* through metabolic engineering

**DOI:** 10.1007/s00253-024-13141-2

**Published:** 2024-04-21

**Authors:** Yun Feng, Yiqi Jiang, Xutong Chen, Li Zhu, Hailong Xue, Mianbin Wu, Lirong Yang, Haoran Yu, Jianping Lin

**Affiliations:** 1 Key Laboratory of Biomass Chemical Engineering of Ministry of Education, College of Chemical and Biological Engineering, Zhejiang University, Hangzhou, 310058 China; 2 Hangzhou Global Scientific and Technological Innovation Center, Zhejiang University, Hangzhou, 311200 China

**Keywords:** Carbamoyltobramycin, Apramycin, Biosynthesis, Lrp/AsnC, Transcriptional regulator, Oxygenase

## Abstract

**Abstract:**

Tobramycin is an essential and extensively used broad-spectrum aminoglycoside antibiotic obtained through alkaline hydrolysis of carbamoyltobramycin, one of the fermentation products of *Streptoalloteichus tenebrarius*. To simplify the composition of fermentation products from industrial strain, the main byproduct apramycin was blocked by gene disruption and constructed a mutant mainly producing carbamoyltobramycin. The generation of antibiotics is significantly affected by the secondary metabolism of *actinomycetes* which could be controlled by modifying the pathway-specific regulatory proteins within the cluster. Within the tobramycin biosynthesis cluster, a transcriptional regulatory factor TobR belonging to the Lrp/AsnC family was identified. Based on the sequence and structural characteristics, *tobR* might encode a pathway-specific transcriptional regulatory factor during biosynthesis. Knockout and overexpression strains of *tobR* were constructed to investigate its role in carbamoyltobramycin production. Results showed that knockout of TobR increased carbamoyltobramycin biosynthesis by 22.35%, whereas its overexpression decreased carbamoyltobramycin production by 10.23%. In vitro electrophoretic mobility shift assay (EMSA) experiments confirmed that TobR interacts with DNA at the adjacent *tobO* promoter position. Strains overexpressing *tobO* with ermEp* promoter exhibited 36.36% increase, and *tobO* with kasOp* promoter exhibited 22.84% increase in carbamoyltobramycin titer. When the overexpressing of *tobO* and the knockout of *tobR* were combined, the production of carbamoyltobramycin was further enhanced. In the shake-flask fermentation, the titer reached 3.76 g/L, which was 42**.**42% higher than that of starting strain. Understanding the role of Lrp/AsnC family transcription regulators would be useful for other antibiotic biosynthesis in other actinomycetes.

**Key points:**

• *The transcriptional regulator TobR belonging to the Lrp/AsnC family was identified*.

• *An oxygenase TobO was identified within the tobramycin biosynthesis cluster*.

• *TobO and TobR have significant effects on the synthesis of carbamoyltobramycin*.

**Supplementary Information:**

The online version contains supplementary material available at 10.1007/s00253-024-13141-2.

## Introduction

*Streptoalloteichus tenebrarius*, also known as *Streptomyces tenebrarius* (Tamura et al. 2008), is an actinomycete that produces carbamoyltobramycin through fermentation. The fermented products are commonly utilized in the industry for producing tobramycin via alkaline hydrolysis (Koch et al. 1973). Tobramycin is an essential and extensively used broad-spectrum antibiotic belonging to the aminoglycoside (Park et al. 2013). It was primarily employed clinically for treating severe infectious diseases caused by Gram-negative bacteria such as *Escherichia coli* and *Pseudomonas aeruginosa* (Wang et al. 2012; Wasserman et al. 2015). Additionally, it exhibited bactericidal effect, even in treating multi-drug resistant microorganisms (MDR) (Pagkalis et al. 2011; Rosalia et al. 2022). The antibacterial mechanism of action involves the binding of tobramycin to the aminoacyl-tRNA recognition site (A-site) on the 30S subunit of bacterial ribosomes, which prevents formation of the normal 70S complex and inhibits protein translation ultimately leading to bacterial death. Furthermore, the deoxygenation at C-3′ reduces the susceptibility to phosphorylation of tobramycin and thereby enhances its efficacy as an antibiotic (Kim et al. 2016).

In early years, traditional mutagenesis breeding techniques such as ultraviolet radiation (UV) and nitrosoguanidine (NTG) were primarily used to obtain high-yielding strains (Qattan and Khattab 2019). However, these traditional breeding methods have limitations of low effectiveness and a demand of an extensive time and labor investment. During the past two decades, various antibiotic biosynthetic gene clusters have been identified (Kudo and Eguchi 2009). The tobramycin biosynthesis gene clusters AJ579650 and AJ810851 and the apramycin biosynthesis gene cluster AJ629123 have been released in National Center for Biotechnology Information (NCBI) database (Kharel et al. 2004; Kudo and Eguchi 2009; Wehmeier and Piepersberg 2009). Genetic modification of the related genes could be carried out based on the analysis of gene clusters and modern molecular biology technology to improve the metabolic and production performance of strains. Firstly, blocking byproduct biosynthesis pathway may increase the proportion of main product in fermentation (Hong and Yan 2012; Ni et al. 2011; Xiao et al. 2014). Secondly, increased copy number of entire biosynthetic gene clusters could also be beneficial to improve secondary metabolite production (Chen et al. 2010; Mitousis et al. 2021). However, since *Streptomyces* have the linear chromosomes and plasmids (Chen et al. 2002), such engineered high-yield strains may be genetically unstable due to the presence of large segments of duplication within the genomes. It was preferable to obtain the strains with comparatively stable fermentation in practical industrial production.

**Fig. 1:**
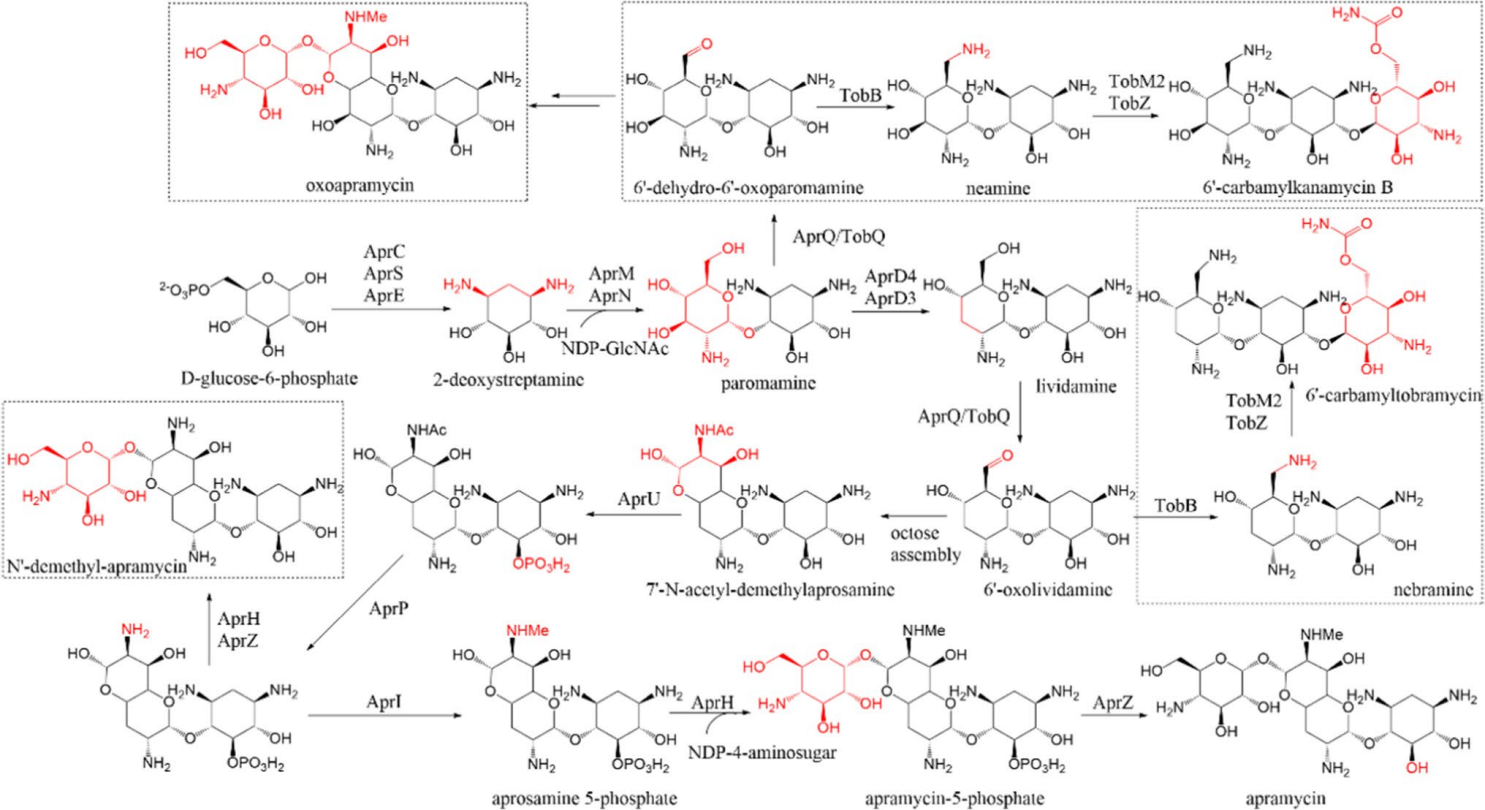
Putative biosynthetic pathways of apramycin, tobramycin, and kanamycin B. The structures highlighted are those undergoing one or more enzymatic catalytic processes

*Streptoalloteichus tenebrarius* Tb used in this study is an industrialized bacterium, mainly producing apramycin, carbamoyltobramycin, and a small amount of carbamoylkanamycin B. Based on the research regarding the aminoglycoside secondary metabolites with 2-deoxystreptamine structure, apramycin, tobramycin, and kanamycin B were all synthesized from the initial substrate D-glucose (Kudo et al. 2021). The key intermediate paromamine was obtained through a series of reactions from D-glucose. Due to the low substrate selectivity of dehydrogenase TobQ, aminotransferase TobB, glucosyltransferase TobM2, and aminoacyltransferase TobZ (Park et al. 2011), there are parallel pathways in the biosynthesis of tobramycin and kanamycin B (Fig. 1) (Ni et al. 2011; Parthier et al. 2012; Tamegai et al. 2002). 6′-Oxolividamine is the last common intermediate splitting the tobramycin and apramycin pathways and is produced from paromamine via sequential dehydration (AprD4), deoxygenation (AprD3), and dehydrogenation (AprQ) reactions (Kim et al. 2016; Kudo et al. 2017; Lv et al. 2016). Then, 7′-N-acetyl-demethylaprosamine with the unique bicyclic octose core structure is synthesized through the aldolase AprG (Fan et al. 2023; Oconnor et al. 1976). To minimize the cytotoxicity, AprU, AprP, and AprI may perform acetylation, phosphorylation, and methylation modifications on intermediates (Sun et al. 2022; Zhang et al. 2022a). Finally, apramycin was synthesized through glycosylation and elimination of phosphate group (Zhang et al. 2021).

With the understanding of synthesis pathways, it is possible to redirect metabolism towards the biosynthesis of tobramycin by knocking out key genes in the apramycin synthesis pathway. However, due to the different metabolic backgrounds of the starting strains, blocking the synthesis of byproduct apramycin may not always produce good results. For example, after knocking out the *aprH-M* gene by Hong (Hong and Yan 2012), the biosynthesis of apramycin was blocked in *Streptomyces tenebrarius* Tt49 and metabolism flowed towards the biosynthesis of carbamoyltobramycin, resulting in a significant increase in the fermentation titer of carbamoyltobramycin. Xiao et al. (2014) knocked out the NDP-octodiose synthase gene *aprK*, and the production of carbamoyltobramycin was increased by 9% in *S. tenebrarius* Tt49, which was different from the 35% of decrease in *Streptoalloteichus tenebrarius* 2444 reported by Mitousis (Mitousis et al. 2021).

The biosynthetic gene cluster of secondary metabolites in *Streptomyces* usually includes structural genes, regulatory genes, and resistance genes. Secondary metabolites synthesis is usually regulated by pathway-specific or global regulatory proteins. The generation of antibiotics can be significantly affected by altering relevant regulatory proteins, especially the pathway-specific regulatory proteins within the cluster (Liu et al. 2019, 2017a, 2021). The leucine-responsive regulatory protein (Lrp/AsnC) family is widespread in bacteria and archaea (Ziegler and Freddolino 2021), which regulates a variety of cellular activities (Brinkman et al. 2003; Peeters and Charlier 2010), such as amino acid metabolism, virulence, motility, nutrient transport, stress tolerance, and antibiotic resistance. The role of Lrp/AsnC family transcription regulators in regulating the biosynthesis of secondary metabolite in the actinomycetes system was still insufficient understanding. It has been reported that Lrp/AsnC family proteins negatively regulate the biosynthesis of erythromycin (Liu et al. 2019, 2017a, 2021) and bitespiramycin (Lu et al. 2019) and positively regulate actinorhodin (Liu et al. 2017b; Yu et al. 2016), thaxtomin A (Liu et al. 2023), and lincomycin (Xu et al. 2020, 2023), while there have been no reports on the metabolic synthesis of aminoglycoside so far.

In this study, a mutant mainly producing carbamoyltobramycin was constructed with apramycin blocked through gene disruption. Due to the presence of common intermediates in the biosynthesis process of apramycin and tobramycin, the regulation of carbon flux through the apramycin and tobramycin pathways appears to be entangled. Blocking the pathway of apramycin may affect the biosynthesis of tobramycin. Due to the differences in the background expression of the producing strains, blocking the reconstruction of metabolic pathways through byproduct biosynthesis may not necessarily lead to completely positive results. So we conducted research on the regulation of tobramycin biosynthesis. We investigated a transcriptional regulator TobR located in the tobramycin biosynthetic gene cluster. The structure of TobR has shown similarity to the Lrp/AsnC family. Gene *tobR* deletion and overexpression strains were constructed to investigate its regulatory role in carbamoyltobramycin production. In vitro electrophoretic mobility shift assay (EMSA) assays verified that TobR directly regulated its neighboring gene *tobO* by interacting with the promoter fragments of *tobO*. Additionally, we constructed two *tobO* overexpression strains with different promoters that both greatly improved the production of carbamoyltobramycin. Finally, the combination of *tobR* disruption and *tobO* overexpression resulted in an engineered strain with a higher yield of carbamoyltobramycin compared with respective *tobR* disruption or *tobO* overexpression strains.

## Materials and methods

### Bacterial strains and general fermentation and growth conditions

Strains in this study are listed in Table 1. The parental strain *S. tenebrarius* Tb (referred as Tb) was obtained from Livzon Pharmaceutical Group Inc. (Guangdong, China). *E. coli* DH5α (TransGen Biotech, China) was used as the host to construct, maintain, and amplify plasmids. *E. coli* BL21(DE3) was used for protein expression (Invitrogen). *E. coli* ET12567/pUZ8002 was a kind gift from Professor Yiling Du (Zhejiang University, China). *E. coli* ET12567/pUZ8002 was used for conjugation to transform plasmids into *S. tenebrarius* strains.

**Table 1:** Strains used in this study

Strains	Relevant characteristic	Source
*E. coli* DH5α	F *recA lac*ZM15	TransGene Biotech, China
*E. coli* BL21(DE3)	*F*^*−*^*omp*ThsdSB (*rB*^*−*^*mB*^*−*^) *gal* dcm (DE#)	Invitrogen
BL21-TobR	*E. coli* BL21(DE3) with the plasmid pET28a-TobR for expression of TobR protein	This study
*E. coli* ET12567(pUZ8002)	*recF dam*- *dcm*- *hsdS cat Km*	(Macneil et al. 1992)
*Streptoalloteichus tenebrarius* Tb (Tb)	Mainly produces carbamoyltobramycin and apramycin	Livzon Pharmaceutical Group Inc. Guangdong, China
Tb -*△aprJ*	Tb with *aprJ* disruption	This study
Tb -*△aprK*	Tb with *aprK* disruption	This study
Tb -*△aprQ*	Tb with *aprQ* disruption	This study
Tb -*△aprM*	Tb with *aprM* disruption	This study
Tb -*△aprI*	Tb with *aprI* disruption	This study
Tb-*△aprJ-△tobR*	Tb-*△aprJ* with *tobR* disruption	This study
Tb-△aprJ/ermEp*-tobR	Tb-*△aprJ* with the plasmid pSpc8660-tobR for overexpression of *tobR*	This study
Tb-*△aprJ/*pSpc8660	Tb-*△aprJ* with the plasmid pSpc8660, as a control strain	This study
Tb-△aprJ/ermEp*-tobO	Tb-*△aprJ* with the plasmid pSpc8660-*tobO* for overexpression of *tobO*	This study
Tb-△aprJ/kasOp*-tobO	Tb-*△aprJ* with the plasmid pSpc-kasOp*-tobO for overexpression of *tobO*	This study
Tb-*△aprJ-△tobO*	Tb-*△aprJ* with *tobO* disruption	This study
Tb-△aprJ-△tobR/ermEp*-tobO	Tb-*△aprJ-△tobR* with the plasmid pSpc8660-*tobO* for overexpression of *tobO*	This study
Tb-*△aprJ-△tobR/*pSpc8660	Tb-*△aprJ-△tobR* with the plasmid pSpc8660, as a control strain	This study

*E. coli* was cultured in LB liquid medium (1% w/v tryptone, 0.5% w/v yeast extract, and 1% w/v NaCl) or on LB agar plates at 37 °C, 220 rpm. All *S. tenebrarius* strains were grown on ISP4 solid medium (BD, USA) for spore preparation or conjugation and in yeast extract-malt extract (YEME, 0.3% w/v yeast extract, 0.3% w/v malt extract, 2.5% w/v sucrose, 0.5% w/v polypeptone and 1% w/v glucose) liquid medium for preparation of genomic DNA and seed medium. The fermentation medium (5% w/v soyabean powder, 1% w/v fish meal, 1% w/v corn flour, 0.8% w/v NH_4_Cl, 0.6% w/v silkworm powder, 0.7% w/v CaCO_3_, 3% w/v soya-bean oil, 0.025% w/v CaCl_2_, and 1.5% w/v glucose) were used for production of carbamoyltobramycin.

For culturing *E. coli* ET12567/pUZ8002 carrying related constructed plasmid used for intergeneric conjugation, antibiotics were supplemented to growth media at the following final concentrations: kanamycin, 25 μg/mL; spectinomycin, 50 μg/mL; and chloramphenicol, 25 μg/mL. For intergeneric conjugation, after co-culturing for about 20 h, spectinomycin and nalidixic acid were coated on ISP4 agar plates at the final concentration of 100 μg/mL and 25 μg/mL, respectively.

### Plasmid construction

All plasmids and primers used in this study are listed in Tables S1 and S2, respectively. The full-length nucleotide sequence of streptomycin 3″-adenylyltransferase (Protein ID: QID24729.1) gene *spc* was codon-optimized for *S. tenebrarius* and ordered from Sangon Biotech (Shanghai, China) as a synthetic DNA, which replaced the gene *aac(3)IV* of vectors pOJ260 (Changsha Yingrun Biotechnology Co., Ltd, Hunan, China) and pIJ8660 (Sun et al. 1999) and constructed the plasmids pSpc260 and pIJ8660-*spc*. Then, nucleotide sequence of promoters ermEp* (Bibb et al. 1994) and kasOp* (Wang et al. 2013) were inserted into the vector pIJ8660-*spc* with the *XhoI* and *BglII* sites, respectively, and constructed the plasmids pSpc8660 and pSpc-kasOp*. Plasmids pSpc260, pSpc8660, and pSpc-kasOp* derived from pOJ260 and pIJ8660 were used for genome editing in *S. tenebrarius.*

The gene cassette *tobR* was amplified from the genomic DNA of Tb using primer pair 28-tobR-F/R. Plasmid vector fragment was amplified from pET28a( +) using primer pair 28a-V-F/R. The cassette fragments were then individually cloned into pET28a( +) using the ClonExpress II one-step cloning kit (Vazyme biotech, Nanjing, China) and confirmed by sequencing, yielding plasmids pET28a-*tobR* for gene expression in *E. coli* BL21(DE3).

Accordingly, the overexpression plasmids pSpc8660-*tobR* and pSpc8660-*tobO* were also constructed as mentioned above, using primer pairs v152-F/R, ermE-tobR-F/R, and ermE-tobO-F/R. The overexpression plasmid pSpc-kasOp*-tobO was constructed by primers V-kasOp-tobO-F/v152-R and tobO-F/ermE-tobO-R.

For deletion of the intergenic region within the gene *aprJ*, two DNA fragments flanking the region were amplified from the genomic DNA of Tb using primer pairs aprJ-F1/R1 and aprJ-F2/R2, respectively. Plasmid vector fragment was amplified from pSpc260 using primer pairs v1139-F/R and connected the homologous arms at both ends through fusion PCR using primer pair aprJ-F1/R2 and then cloned into pSpc260 generating the disruption plasmid pSpc260-*△aprJ*. Besides, the disruption plasmid pSpc260-*△aprK*, pSpc260-*△aprQ*, pSpc260-*△aprI*, pSpc260-*△aprM*, pSpc260-*△tobR*, and pSpc260-*△tobO* constructed as mentioned above, using primer pairs aprK-F1/R1/F2/R2, aprQ-F1/R1/F2/R2, aprI-F1/R1/F2/R2, aprM-F1/R1/F2/R2, tobR-F1/R1/F2/R2, and tobO-F1/R1/F2/R2.

### Construction of *S. tenebrarius* strains

For gene deletion, suicide plasmids pSpc260-*△aprJ*, pSpc260-*△aprK*, pSpc260-*△aprQ*, pSpc260-*△aprI*, pSpc260-*△aprM*, pSpc260-*△tobR*, and pSpc260-*△tobO* derived from pOJ260 were transformed into *E. coli* ET12567/pUZ8002 and then introduced into *S. tenebrarius* by conjugation. Single crossover recombination strains were selected by culturing the transformants on ISP4 plates which containing 100 μg/mL spectinomycin at 37 °C. Subsequently, after three rounds of sporulation on plates without antibiotics, double crossover mutants were selected (Figs. S1, S2, and S3). To verify the genotype of double exchange strains, primer pairs di-aprJ-F/R, di-aprK-F/R, di-aprQ-F/R, di-aprI-F/R, di-aprM-F/R, di-tobR-F/R, and di-tobO-F/R were used for PCR. Name the successfully constructed strains as Tb-*△aprJ*, Tb-*△aprK*, Tb-*△aprQ*, Tb-*△aprI*, Tb-*△aprM*, Tb-*△aprJ-△tobR*, and Tb-*△aprJ-△tobO.*

The overexpression plasmids pSpc*8660-tobR*, pSpc8660*-tobO*, and pSpc-kasOp*-tobO were introduced into *S. tenebrarius* as mentioned above. To get the overexpression strains, exconjugants were selected on ISP4 agar plates supplemented with spectinomycin and identified by PCR. These overexpression strains were named as Tb-△aprJ/ermEp*-tobR, Tb-△aprJ/ermEp*-tobO, Tb-△aprJ/kasOp*-tobR, and Tb-△aprJ-△tobR /ermEp*-tobO, respectively*.* The empty vector pSpc8660 was also transferred into Tb-*△aprJ* and Tb-*△aprJ-△tobR* to generate control strains named Tb-*△aprJ*/pSpc8660 and Tb-*△aprJ-△tobR*/pSpc8660.

### Heterologous expression and purification of TobR

For heterologous expression of TobR protein in *E. coli* BL21(DE3), the *tobR* was amplified by PCR from the genome of Tb with the primer pair 28-tobR-F/R. Then, it was cloned into pET28a and generating an expression plasmid with N-terminal His-tag fusion. The constructed plasmid pET28a-tobR was introduced into *E. coli* BL21(DE3), and the protein expression was induced with IPTG at a final concentration of 0.1 mM at 30 °C for 10 h. His_6_-tagged TobR protein was extracted and purified on a Ni^2+^-NTA spin column (Shenggong). The quality of the purified protein was estimated by sodium dodecyl sufate polyacrylamide gel electrophoresis (SDS-PAGE). The protein concentration was measured by Pierce BCA Protein Assay Kit (Shanghai, Thermo Fisher Scientific Co., Ltd).

### Electrophoretic mobility shift assays (EMSAs)

The EMSAs were performed as described previously (Hellman and Fried 2007). PCR was performed using Tb-*△aprJ* genome as a template and ptobO-F/R primers to obtain the fragment named as *P*_*tobO*_ probe, which is the intergenic sequence between *tobR* and *tobO*. Moreover, the promoter regions of each transcriptional unit in the tobramycin biosynthesis cluster were amplified by PCR with 7 pairs of primers, including ptobE-F/R, ptobT-F/R, ptobB-F/R, ptobZ-F/R, ptobS1-F/R, ptobM1-F/R, and ptobA-F/R, respectively (Table S2). These probes were named *P*_*tobE*_, *P*_*tobT*_, *P*_*tobB*_, *P*_*tobZ*_, *P*_*tobS1*_, *P*_*tobM1*_, and *P*_*tobA*_. The 100 ng DNA probes were incubated individually with various concentrations of His_6_-tagged TobR in binding buffer (10 mL pH 7.5 Tris–HCl, 5 mM MgCl_2_, 60 mM KCl, 10 mM DTT, 50 mM EDTA, and 10% glycerol) at 30 °C for 20 min in 20 μL reaction mixture. After incubation, the samples were fractionated on 6% native PAGE gels in ice-cold 0.5 × TBE buffer at 100 V for 120 min.

### Fermentation

The strains were cultured on MS agar plates for about 5–7 days at 37 °C for sporulation. The spores were then inoculated into 30 mL YEME medium (seed medium) in 250-mL flasks and cultured at 37 °C, 220 rpm for 20 h. The seed culture was then inoculated into the 30 mL fermentation medium giving a 3% vaccination dose and then cultured at 37 °C, 220 rpm for 144 h.

### Quantitative analysis

Dilute the supernatant to a certain ratio and derivatize it with 2% 2,4-dinitrofluorobenzene and then filter it through a Millipore membrane. The aminoglycosides antibiotics were analyzed as previously described (Barends et al. 1987) with some modification. For the analysis of tobramycin, culture samples from fermentation were centrifuged at 10,000 g for 5 min to remove the mycelia; the supernatants were derivatized with 2% 2,4-dinitrofluorobenzene and filtered through a Millipore membrane (pore diameter, 0.22 μm). Samples were analyzed by high-performance liquid chromatography (HPLC) through the C-18 column (Hypersil BDS 5 μm, 4.6 mm × 250 mm), with a UV detector at 365 nm. A 0.01 mM ammonium acetate aqueous solution (the pH adjusted with phosphoric acid to 4.0)/acetonitrile (47: 53, v/v) was used as the mobile phase with an elution rate of 1 mL/min. All results were reported as the average of biological triplicates.

### RNA preparation and qRT-PCR assay

Cells of Tb-*△aprJ*, Tb-△aprJ/ermEp*-tobO, and Tb-△aprJ/kasOp*-tobO grown in fermentation medium for 24 h, 72 h, and 120 h were harvested by centrifugation. Total RNA was collected using the EASYspin Plus RNA extraction/purification kit (Aidlab Biotechnologies Co., Ltd). The integrality and quantity of the RNA were detected by 1% agarose gel electrophoresis and a microplate reader. RNA samples were treated by reverse transcription using the ReverTra AceTM qPCR RT Master Mix with gDNA Remover kit (TOYOBO). The obtained cDNAs were used as templates for qPCR. qPCR was employed on the TransStart Top Green qPCRSuperMix (TransGen Biotech) using corresponding primers listed in Table S2. A 20 μL reaction mixture contained 10 μL 2 × TransStart Top Green qPCRSuperMix, 0.4 μL cDNA, 0.4 μL per primer (about 0.2 μM), and 8.8 μL RNase-free ddH_2_O. The running conditions were 95 °C for 10 min (step 1), 30 cycles of 94 °C for 10 s, 56 °C for 10 s, and 72 °C for 10 s (step 2). Each experiment was carried out with three independent biological replicates and three experimental replicates. As its constant transcriptional level between Tb-*△aprJ* and its derivatives, the endogenous *gapA* gene was used as an internal control to normalize samples.

### Bioinformatics analysis

Online tools are available for protein homology analysis, multiple sequence alignment and structure prediction. The BLAST search engine provided by the National Center for Biotechnology Information (https://blast.ncbi.nlm.nih.gov/Blast.cgi) was used for protein homology analysis. Online comparison tool CLUSTALW online sequence alignment tool (https://www.genome.jp/tools-bin/clustalw/) was used for multiple sequence alignment. The ΑlphaFold 2 (https://colab.research.google.com/github/sokrypton /ColabFold/blob/main/ΑlphaFold2.ipynb) was used to predict the structure of TobR and TobO.

## Results

### Elimination of byproduct apramycin

**Fig. 2:**
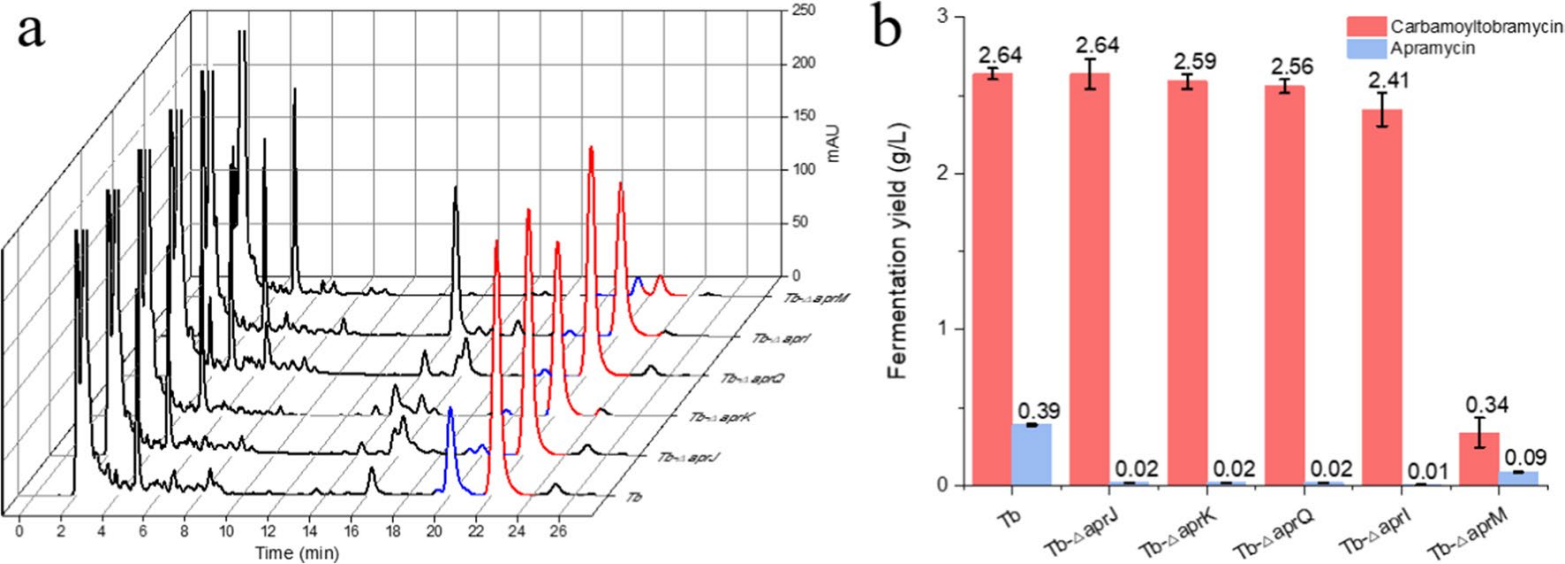
Elimination of apramycin in *S. tenebrarius.* **a** HPLC chromatogram of Tb and mutants. The blue curve represents the peak of apramycin, while the red curve represents the peak of carbamoyltobramycin. **b** Fermentation products of Tb and mutants analyzed by HPLC. Mean values of 3 replicates are shown, with the standards indicated by error bars

To investigate the effect of blocking the biosynthesis of byproduct apramycin on the production of carbamoyltobramycin, we knocked out several key enzymes in the biosynthesis pathway of apramycin including putative phosphosugar mutase AprJ, NDP-octose synthase AprK, aminoglycoside 6′-dehydrogenase AprQ, N-methyltransferase AprI, and glycosyltransferase AprM. As shown in Fig. 2a, in comparison with the parent strain Tb, Tb-*△aprJ*, Tb-*△aprK*, Tb-*△aprQ*, and Tb-*△aprI* showed comparable biosynthesis of carbamoyltobramycin, while the production of apramycin was significantly decreased to less than 1% of the production of carbamoyltobramycin. However, the biosynthesis of tobramycin in Tb-*△aprM* was severely impacted with the production of carbamoyltobramycin decreased to 0.34 g/L, only 12.8% of the initial strain (2.64 g/L), and the byproduct apramycin was still synthesized although its yield (0.09 g/L) was decreased to 23% of the original level (0.39 g/L) (Fig. 2b). Gene *aprM* putatively encoding glycosyltransferase might be functionally similar to TobM1, a putative aminoglycoside 4-glucosaminyltransferase, or TobM2, a putative 6-glucosyltransferase, which participated in tobramycin and apramycin pathway (Fig. S4 and table S3). In addition, the Tb-*△aprQ* and Tb*-△aprI* mutants showed new peaks on the HPLC spectrum. Due to the blockage of the biosynthetic pathway of apramycin, there may be an accumulation of intermediate products, and the new peak appeared after the *aprQ* was knocked out could be lividamine or 6′-dehydro-6′-oxoparomamine (Wang et al. 2021). Furthermore, the new compound appearing in the Tb-*△aprI* strain might be N’-demethyl-apramycin (Zhang et al. 2022a). As for Tb-*△aprJ* and Tb-*△aprK,* the fermentation of carbamoyltobramycin was not affected, and the byproduct was almost completely removed. And there was no significant changes observed in the process of cell growth and spore synthesis for Tb-*△aprJ* and Tb-*△aprK*. The mutant Tb-*△aprJ* was selected for further research due to its slightly higher production of 2.64 g/L carbamoyltobramycin compared with 2.59 g/L of Tb-*△aprK*. Overall, blocking the biosynthesis of apramycin in this work did not have a significant impact on the production of carbamoyltobramycin. Actually, the effect of blocking apramycin synthesis on production of carbamoyltobramycin was strain-specific. For example, by blocking the biosynthesis of apramycin in strains *S. tenebrarius* Tt49, the production of carbamoyltobramycin was increased by sixfold in the study of Hong (Hong and Yan 2012) and by 9% in the study of Xiao (Xiao et al. 2014). However, Lena Mitousis et al. found that blocking the biosynthesis of apramycin decreased the production of carbamoyltobramycin by 35% in *S. tenebrarius* 2444 (Mitousis et al. 2021). We found that the starting strains *S. tenebrarius* Tt49 that Xiao and Hong used mainly produced apramycin, while the proportion of apramycin and carbamoyltobramycin in *S. tenebrarius* 2444 that Lena Mitousis used was similar. And the proportion of apramycin was only 14.8% of carbamoyltobramycin in our starting strain *S. tenebrarius* Tb. Hence, effect of knocking out the gene of apramycin biosynthesis pathway on the carbamoyltobramycin synthesis would be strain-specific.

### Bioinformatics analysis of TobR in *S. tenebrarius*

With the strain eliminating the production of main byproduct, we attempted to improve the yield of carbamoyltobramycin in the engineered strains by transcriptional regulation reconstruction. After analyzing the whole tobramycin biosynthesis gene cluster (Table S3), we found a gene *tobR* that may encode a transcriptional regulator TobR, and no other genes in the cluster may encode transcriptional regulators. According to the prediction results of BlastP, TobR may belong to the the Lrp/AsnC family. We first carried out a multiple sequence alignment between TobR and other Lrp/AsnC family members. TobR exhibited about 20% sequence identity with most of previously reported Lrp homologs in PDB database, with the highest similarity with the Lrp/AsnC of *E. coli*, reaching 29.37% (Fig. S5). Furthermore, TobR showed high sequence similarity of 68.89–88.27% with its homologues from actinomycetes such as *Streptoalloteichus hindustanus* (WP_073480789.1), *Actinokineospora alba* (WP_228769743.1), *Alloactinosynnema sp*. L-07 (CRK55752.1), *Nonomuraea* sp. KC401 (WP_138203008.1), and *Kribbella antibiotica* (WP_138203008.1) (Fig. S6). Therefore, the understanding of TobR function in this study might be useful for illustrating the role of Lrp/AsnC family transcription regulators in other actinomycetes.

**Fig. 3:**
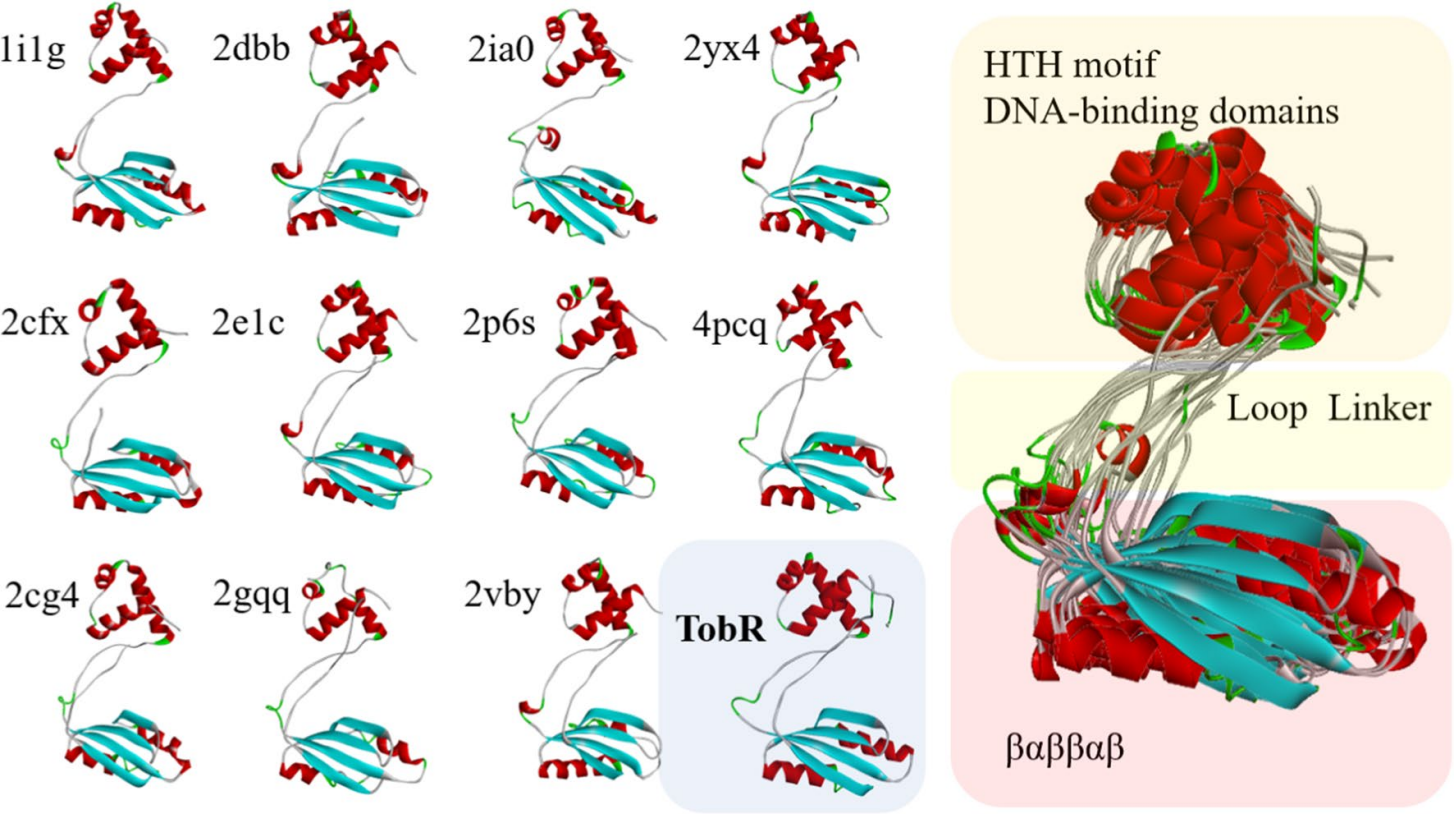
Structural analysis of TobR protein. The structure of TobR was predicted by Alphafold 2, while other protein structures belongs to Lrp/AsnC family have been reported in the PDB database (PDB ID: 1i1g, 2dbb, 2ia0, 2yx4, 2cfx, 2e1c, 2p6s, 4pcq, 2cg4, 2gqq, and 2vby)

Although the Lrps homologues from different sources exhibited low sequence conservation characteristics (Kawashima et al. 2008), their structure was highly similar (Peeters and Charlier 2010). As shown in Fig. 3, it typically consists of two domains including one N-terminal DNA binding structure with a common helix-turn-helix folding (HTH motif) and the other with a typical αβ-ligand binding domain of sandwich folding at C-terminal (Ettema et al. 2002). These two domains are linked by a flexible loop with a length of approximately 15 amino acids (de los Rios and Perona 2007; Reddy et al. 2008). In the regulation process of biological reaction, Lrps proteins normally function with status of multimer including dimer, tetramer, hexamer, octamer, and dodecamer that are usually observed for other Lrp/AsnC family transcription regulators (Brinkman et al. 2000; Koike et al. 2004; Leonard et al. 2001; Pritchett et al. 2009). The transition of different association conformations may influence the interaction effect between DNA and protein (Jeong et al. 2015). The structural model of TobR was predicted using Alphafold 2, and the plDDT score indicates the high prediction accuracy (Fig. S7). Then, the structure of TobR was compared with the structures of other Lrp/AsnC family transcriptional regulators reported in the PDB database (Fig. 3). TobR exhibits the Lrp/AsnC family structural feature that HTH motif is linked to the αβ-ligand binding domain through a flexible loop, suggesting that TobR belongs to Lrp/AsnC family.

### Inactivation and overexpression of *tobR* in *S. tenebrarius*

**Fig. 4:**
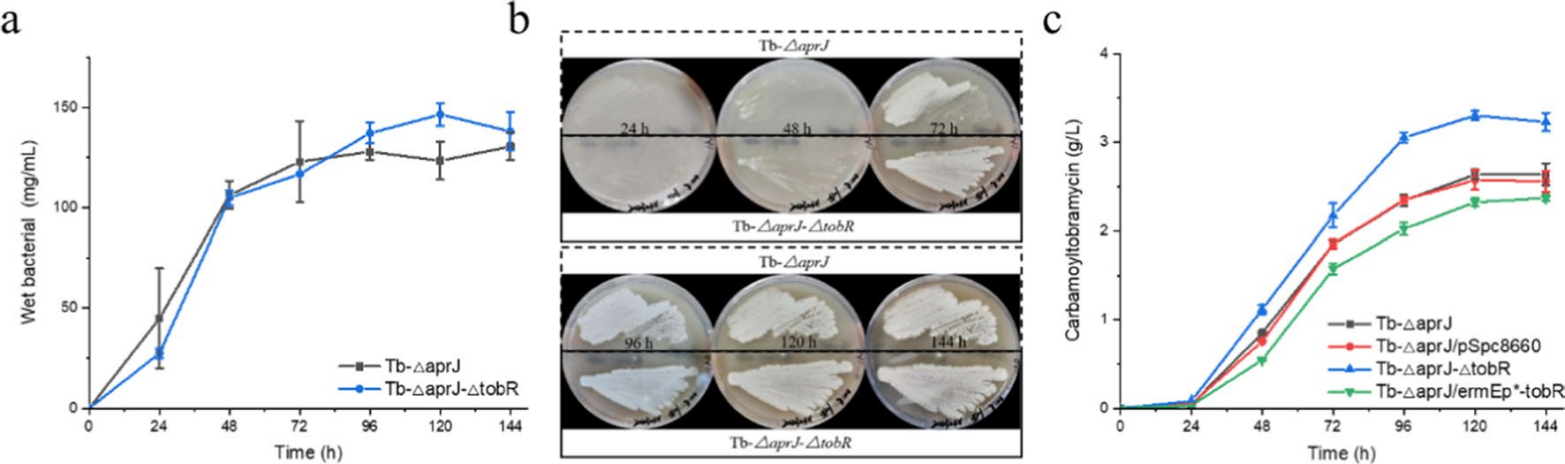
The impact of TobR on *S. tenebrarius.* **a** Accumulation of biomass in liquid culture of Tb-*△aprJ* and Tb-*△aprJ-△tobR.* **b** Growth state of Tb-*△aprJ* and Tb-*△aprJ-△tobR* in MS solid medium. **c** Carbamoyltobramycin production of *tobR* disruption and overexpressed strains analyzed by HPLC. Mean values of 3 replicates are shown, with the standards indicated by error bars

The mutant strain Tb-*△aprJ-△tobR* and the starting strain Tb-*△aprJ* were carried out to investigate the impact of TobR on *S. tenebrarius* growth. The cells were collected and weighted during the fermentation process, and the growth and sporulation of each strain were observed during the cultivation on MS solid medium at 37 °C. There was no significant difference in wet bacterial mass and mycelial growth or spore formation between the two strains, indicating that TobR may not play a role in regulating mycelial growth and morphological differentiation (Fig. 4a&4b).

The strains Tb-*△aprJ*, Tb-*△aprJ-△tobR*, Tb-*△aprJ*/ermEp*-tobR, and their control strain Tb-*△aprJ*/pSpc8660 were also fermented in shake flasks to investigate the production yield. As shown in the Fig. 4c, in comparison with the parent strain Tb*-△aprJ*, Tb-*△aprJ-△tobR* improved the production of carbamoyltobramycin by 22.35%, from 2.64 to 3.23 g/L. However, overexpression of the *tobR* in Tb*-△aprJ* reduced the production of target product by 10.32%, from 2.56 to 2.37 g/L. These results suggested that the Lrp/AsnC family transcription regulator TobR might have a negative effect on the carbamoyltobramycin biosynthesis in *S. tenebrarius*.

### qRT-PCR and EMSA analysis of TobR

**Fig. 5:**
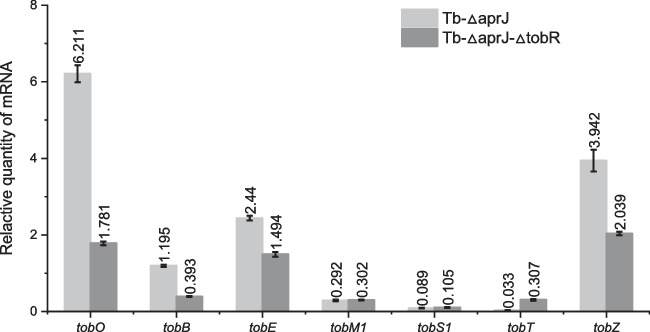
Changes in transcription levels of related genes after knocking out *tobR* at 72 h in fermentation

The yield of carbamoyltobramycin was increased in TobR knockout strains, suggesting that TobR negatively regulates the biosynthesis of tobramycin. qRT-PCR assay was performed in further investigate the impact of TobR at 72 h in fermentation. As shown in Fig. 5, the transcription levels of the adjacent gene *tobO* and each transcription unit related to the tobramycin biosynthetic pathway were compared in Tb-*△aprJ* and Tb-*△aprJ*-*△tobR* strains. The transcription levels of the transcription units *tobO*, *tobB*, *tobE*, and *tobZ* in the Tb-*△aprJ*-*△tobR* significantly decreased compared with the control group Tb-*△aprJ*, while the changes in *tobS1* and *tobM1* were not significant. Moreover, the genes involved in these transcription units were all speculated to be genes in the tobramycin biosynthesis pathway. Furthermore, the transcription level of *tobT*, which is presumed to encode the transporter (Fig. 7f), increased by 9.3-fold compared to the original level.

**Fig. 6:**
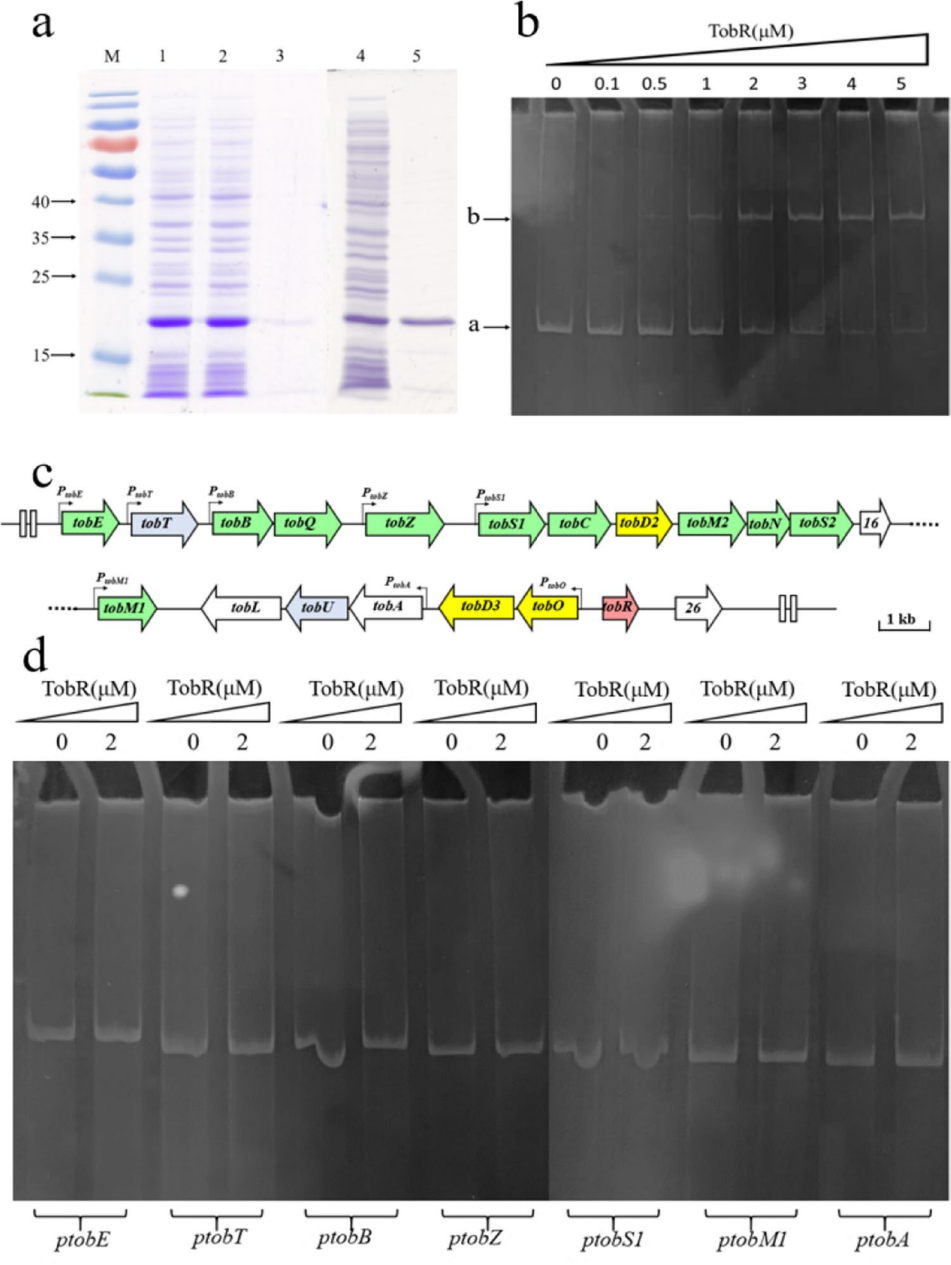
EMSA Analysis of TobR and *P*_*tobO*_*.* **a** SDS-PAGE of TobR. M, protein ladder; (1) whole cell; (2) supernatant; (3) precipitation; (4) crude protein/supernatant; (5) pure protein/TobR. **b** EMSA analysis of TobR binding to *P*_*tobO*_, (a) *P*_*tobO*_ probe; (b) TobR-*P*_*tobO*_ complex.** c** Transcription unit of tobramycin biosynthetic gene cluster. *P*_*tobE*_,* P*_*tobT*_, *P*_*tobB*_, *P*_*tobZ*_, *P*_*tobS1*_, *P*_*tobM1*_, *P*_*tobA*_, and *P*_*tobO*_ represented probes of promoter regions of each transcription unit. The genes highlighted in green in the cluster were speculated to be related to the tobramycin biosynthesis pathway. The genes highlighted in blue were speculated to encode transport proteins. The white markers represent proteins with unknown functions in tobramycin biosynthesis, and the red markers represent potential transcriptional regulatory factors. **d** The regulatory targets of TobR on tobramycin biosynthesis gene cluster analyzed by EMSA

Due to the inability to summarize patterns from changes in transcription levels of relevant genes, we further confirmed the mechanism of TobR through EMSA analysis. We expressed His6-tagged TobR in *E. coli* BL21(DE3) and examined its affinity to *P*_*tobO*_ (*tobR-tobO-int*) with EMSA. With the induction of IPTG, BL21-TobR effectively expressed the target protein with a size of 21.5 kDa, consistent with the predicted size of TobR-His_6_ fusion protein (Fig. 6a). The fusion protein was well-expressed in soluble form and mainly distributed in the supernatant without formation of inclusion bodies. Purification through a Ni^2+^-NTA spin column from the supernatant yielded pure TobR protein (Fig. 6a). A TobR-*P*_*tobO*_ complex formed in a concentration-dependent manner was observed, and the higher the concentration of TobR added, the more complex formed (Fig. 6b).

Besides the interaction between TobR and its neighboring genes, we created 500 bp DNA probes containing promoter regions for various transcription units within the gene cluster to investigate whether TobR directly regulates synthetic genes by analyzing their DNA–protein binding status in EMSA experiments (Fig. 6c). Under the 2 μM concentrations of TobR, there was no obvious binding between TobR and each probe (Fig. 6d). Further, improve the protein concentrations of TobR to 5 μM, but no binding was found (Fig. S8), indicating that there was no direct regulation relationship between TobR and genes within the tobramycin biosynthesis gene cluster.

### Bioinformatics analysis of TobO

**Fig. 7:**
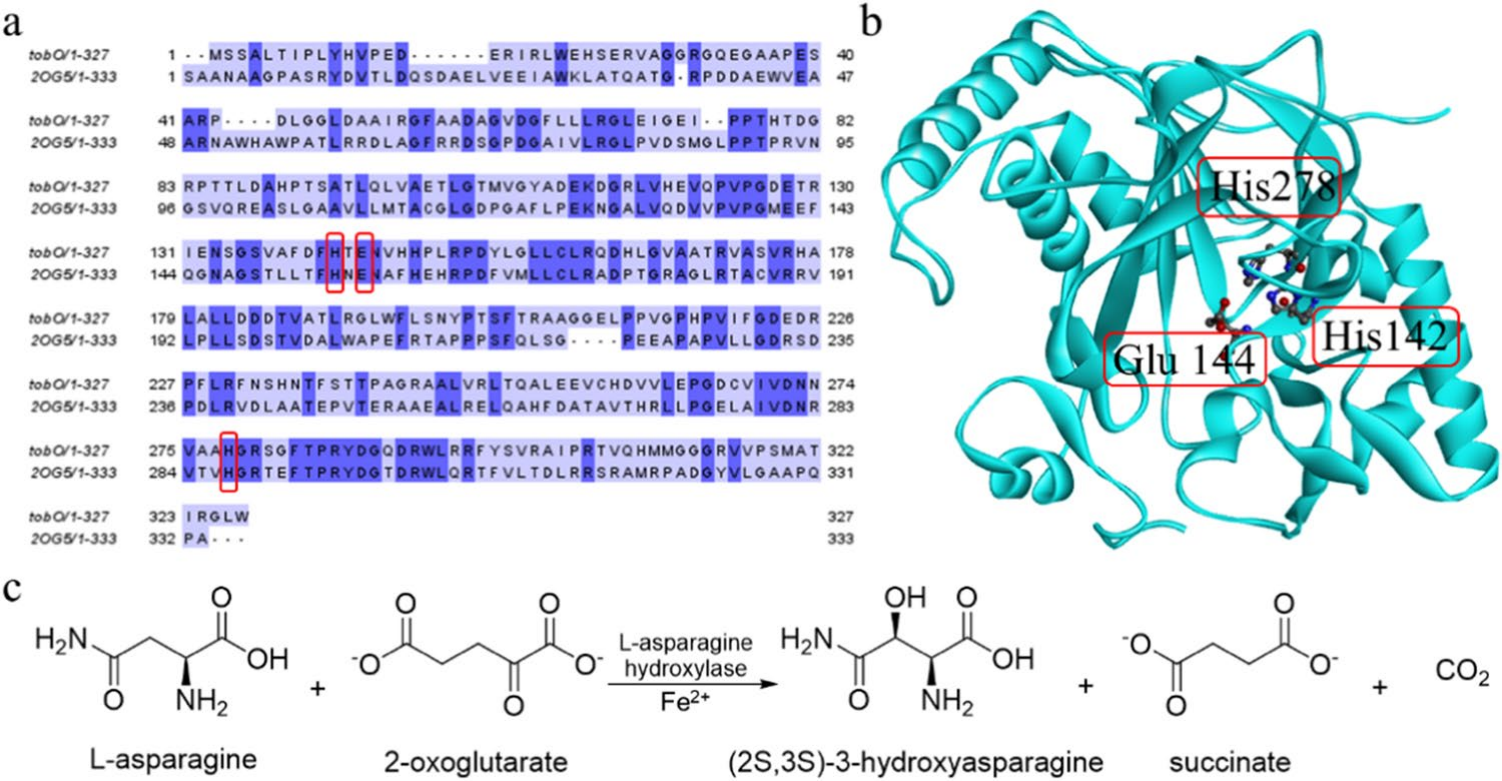
Bioinformatics analysis of TobO. **a** The amino acid sequences of TobO and its homologous proteins (blue background indicating the protein percent identity, the red box labeled amino acid represents the catalytic triad formed with ferrous ion; asparagine oxygenase PDB ID: 2OG5 from *Streptomyces coelicolor* has a protein similarity of 47.0% with TobO). **b** The structure of TobO predicted by the online tool Αlphafold 2. **c** Catalytic reaction of L-asparagine hydroxylase

According to the qRT-PCR and EMSA results of TobR, the Lrp/AsnC family regulator TobR might affect the synthesis of tobramycin by directly acting on its adjacent gene *tobO* (Fig. 6). We then investigated the involvement of TobO in the biosynthesis of carbamoyltobramycin in *S. tenebrarius* using bioinformatics tools. The *tobO* is a 984 bp gene encoding a protein TobO consisting of 327 amino acids. A sequence alignment was performed with the sequence of TobO as input using the tool of protein blast in the NCBI database. The results showed that TobO possessed a sequence identity of 71.33% and 70.32% with TauD/TfdA family dioxygenase from *Actinokineospora diospyrosa* (WP_253887134.1) and *Actinokineospora* sp. PR83 (WP_236229521.1), respectively. Additionally, it showed a sequence identity of 60.32% with L-asparagine oxygenase from *Streptomyces rubradiris* (GHH24494.1) suggesting that *tobO* might encode an α-ketoglutarate-dependent non-haem iron enzyme. Sequence and structure alignment were then performed between TobO and the known structure asparagine oxygenase (PDB ID: 2OG5) from *Streptomyces coelicolor* (Strieker et al. 2007), showing a sequence similarity of 47.0% (Fig. 7a). It was determined that His142, Glu144, and His278 were the catalytic triad of TobO, which formed active center with ferrous ion (Fig. 7b). Generally, L-asparagine oxygenase is responsible for the oxidation of L-asparagine and converting α-ketoglutarate into succinate and CO_2_ (Fig. 7c). Based on the sequence analysis and structural characteristics of TobR and L-asparagine oxygenase, TobO might be an amino acid oxidase, responsible for the related metabolic reactions of amino acids in the organism.

### Inactivation and overexpression of *tobO* in *S. tenebrarius*

**Fig. 8:**
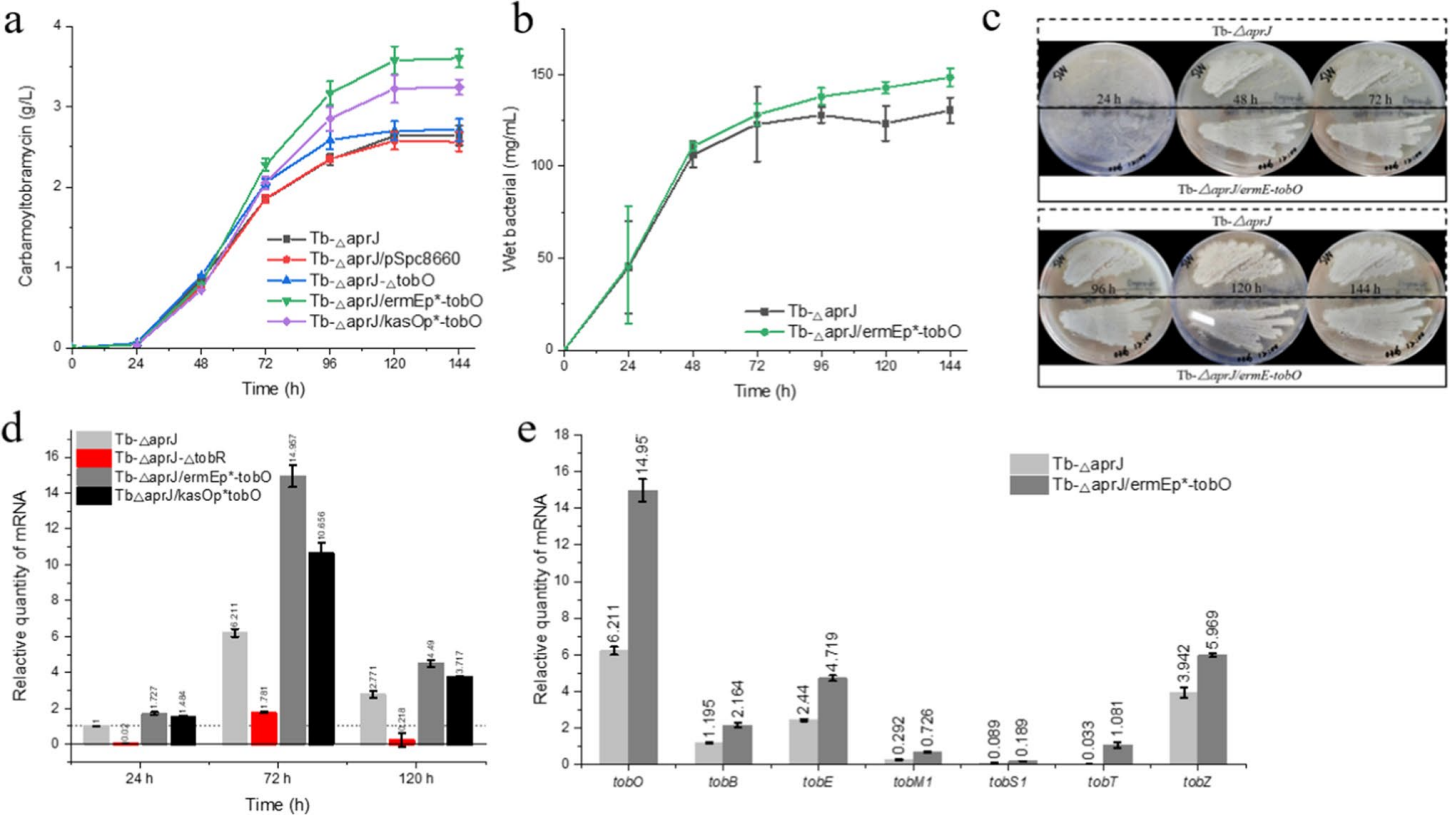
The impact of TobO on *S. tenebrarius.* **a** Carbamoyltobramycin production of *tobO* disruption and overexpressed strains by HPLC analysis. **b** Accumulation of biomass in liquid culture of Tb-*△aprJ* and Tb-△aprJ/ermE*p-tobO. **c** Growth state of Tb-*△aprJ* and Tb-△aprJ/ermE*p-tobO in MS solid medium. **d** Transcriptional analysis of the *tobO* in Tb-*△aprJ*, Tb-△aprJ/ermEp*-tobO, and Tb-△aprJ/kasOp*-tobO at 24 h, 72 h and 120 h in fermentation. **e** Transcriptional analysis of the *tobB*, *tobE*, *tobM1*, *tobS1*, *tobT*, and *tobZ* in Tb-*△aprJ* and Tb-△aprJ/ermEp*-tobO at 72 h in fermentation. Mean values of 3 replicates are shown, with the standards indicated by error bars

To explore the effect of *tobO* on carbamoyltobramycin production, the shake flask fermentation yield of starting strain Tb-*△aprJ*, overexpression strains Tb-△aprJ/ermEp*-tobO, Tb-△aprJ/kasOp*-tobO, a knockout strain Tb-*△aprJ-△tobO*, and an empty vector control Tb-*△aprJ*/pSpc8660 were measured. The titer of carbamoyltobramycin was increased by 36.36% and 22.84% in the overexpression strains ermEp*-tobO (3.60 g/L) and kasOp*-tobO (3.24 g/L) compared with the starting strain Tb-*△aprJ* (2.64 g/L), while there was no significant change in Tb-*△aprJ-△tobO* (2.71 g/L) (Fig. 8a). The biomass during fermentation were also analyzed, and the wet cell cumulant of Tb-*△aprJ*/ermEp*-tobO (0.148 g/mL) was slightly higher than that of starting strain Tb-*△aprJ* (0.135 g/mL) (Fig. 8b), indicating that the overexpression of *tobO* might promote the growth of strains during the fermentation process. However, there was no significant difference observed in the sporulation status in MS solid medium between Tb-*△aprJ*/ermEp*-tobO and Tb-*△aprJ-△tobO* (Fig. 8c).

In order to further determine the role of *tobO*, we measured the transcripts of *tobO* at mycelial growth (24 h), product accumulation (72 h), and stabilization stage (120 h) during the fermentation process in Tb-*△aprJ*, Tb-△aprJ/ermEp*-tobO, and Tb-△aprJ/kasOp*-tobO by qRT-PCR. The transcriptional level of *tobO* in Tb-*△aprJ* after 72 h and 120 h of growth was increased by 6.2- and 2.8-folds, respectively, compared with 24 h (Fig. 8d), indicating that *tobO* may play a role in the synthesis of secondary metabolites. In addition, the transcripts of *tobO* in overexpressed strains Tb-△aprJ/ermEp*-tobO and Tb-△aprJ/kasOp*-tobO at 72 h was increased by 2.4- and 1.7-folds respectively compared with in Tb-*△aprJ*, suggesting that the overexpression of *tobO* did improve the transcripts of *tobO*. However, ermEp* showed higher promoter activity compared with kasOp* in this experiment, different from previous literature (Wang et al. 2013). The different bacterial species may be the key reason. The strain used in this experiment was *Streptoalloteichus tenebrarius*, belonging to *Pseudoocardiaceae*, while the *Streptomyces coelicolor*, *Streptomyces venezuelae*, and *Streptomyces avermitilis* in which the kasOp* was tested belong to *Streptomycetaceae* (Bai et al. 2015; Dong et al. 2020; Myronovskyi and Luzhetskyy 2016).

Moreover, both knocking out *tobR* and overexpressing *tobO* increased the fermentation titer of carbamoyltobramycin, and TobR could directly interact with DNA fragments of the *P*_*tobO*_ intergenic region. *tobO* was a possible target gene regulated by TobR, which negatively regulates biosynthesis of tobramycin by inhibiting transcriptional expression of *tobO*. However, the knockout of the *tobO* did not have a significant impact on the production of carbamoyltobramycin (Fig. 7a). The transcripts of *tobO* during fermentation of Tb-*△aprJ* and Tb-*△aprJ*-*△tobR* strains indicate that the transcription level of *tobO* was rapidly decreased by 0.02-, 0.29- and 0.08-folds compared with Tb-*△aprJ* at 24 h, 72 h, and 120 h, respectively, after blocking *tobR*, which demonstrate that TobR has a positive regulatory effect on the expression of the *tobO*.

In addition, the transcription status of each transcription unit in the tobramycin biosynthesis gene cluster of Tb-△aprJ/ermEp*-tobO was also measured. As shown in Fig. 7e, the transcription levels of all transcription units were significantly increased in the overexpressing strains. Among them, *tobT* showed the highest increase in transcription levels, which was 32.8-fold of the original level.

### Combination of *tobR* disruption and *tobO* overexpression

**Fig. 9:**
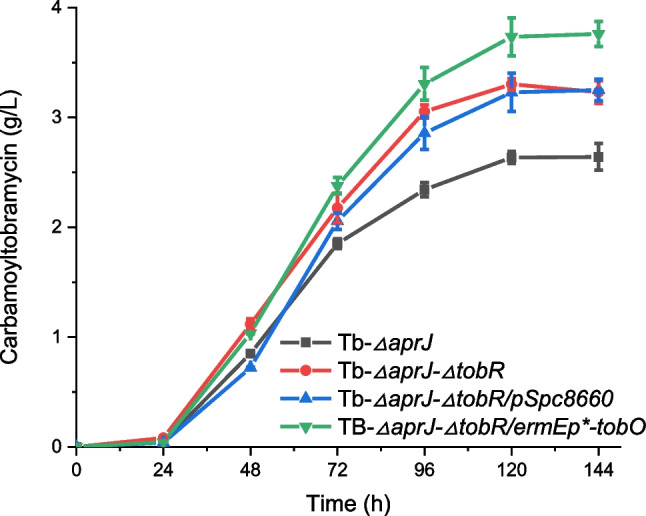
Carbamoyltobramycin production measured by HPLC analysis. Mean values of 3 replicates are shown with the standard deviation as the error bars

Both *tobR* knockout and *tobO* overexpression promoted the fermentation production of carbamoyltobramycin. We hence wondered if the additive effect could be achieved by combining *tobR* knockout and *tobO* overexpression. Production of carbamoyltobramycin was detected in shake flask fermentation after overexpressing the *tobO* following the knockout of the *tobR*. The mutant strain Tb-△aprJ-△tobR/ermEp*-tobO produced carbamoyltobramycin with a higher titer compared with strains with single modifications. The shaking flask fermentation level of this strain reached 3.76 g/L (Fig. 9), a significant increase of 42.42% compared with the Tb-*△aprJ* strain. The results suggested that the combination of *tobR* knockout and *tobO* overexpression showed a beneficially additive impact on the carbamoyltobramycin biosynthesis.

## Discussion

In this study, the mutant strain Tb-*△aprJ* mainly producing carbamoyltobramycin was first selected with the main byproduct apramycin completely blocked by gene disruption of *aprJ*. The elimination of fermentation by-product from industrial production strain can significantly improve product quality while reducing the input of production costs during the purification process.

Intriguingly, a novel Lrp-like protein TobR was identified from tobramycin biosynthesis cluster of *S. tenebrarius* with bioinformatics analysis. The gene *lrp* is typically found in close proximity to its target gene (Peeters and Charlier 2010). For example, in *Sulfolobus solfataricus* (Song et al. 2013), LysM directly regulates the adjacent *lysWXJK* operon. In *Halobacterium salinarum* R1 (Schwaiger et al. 2010), LrpA1 inhibits its neighboring *aspB3*. SACE_Lrp in *Saccharopolyspora erythraea* suppresses the transcription level of its adjacent gene *SACE_4838* and affects erythromycin biosynthesis (Liu et al. 2017a). Additionally, the Lrp/AsnC family transcription regulator SCO3361 found in *Streptomyces coelicolor* directly regulates the expression of its neighboring gene *SCO3362* (Liu et al. 2017b). The similarity among the Lrp/AsnC family implies that TobR may directly bind with *tobR-tobO-int* and control the expression of *tobO*. As expected, through the EMSA analysis of TobR, we found it directly combined with the promoter region of the *tobO* operon rather than other transcription units in tobramycin biosynthesis cluster. Meanwhile, disruption of *tobR* and overexpression of *tobO* increased carbamoyltobramycin production by 22.34% (3.23 g/L) and 36.36% (3.60 g/L), respectively, compared with the parent strain Tb-*△aprJ*.

Furthermore, this work reveals that the transcription of *tobO* is positively regulated by TobR, but TobR may negatively regulate the biosynthesis of tobramycin through other regulation, and the regulatory mechanism of TobR still remains questionable. The TobR in this study may differ from the Lrp/AsnC family transcription regulatory factors reported in the literature that directly inhibit the expression of adjacent genes (Liu et al. 2019). The increase in *tobO* transcription level can significantly promote the biosynthesis of tobramycin, while the knockout of *tobO* did not have an inhibitory effect. Therefore, the increase of tobramycin synthesis caused by the knockout of *tobR* was likely due to the presence of other regulated key genes throughout the entire genome in *S. tenebrarius*. TobR may have other binding sites outside of the tobramycin biosynthesis gene cluster range. For example, SSP_Lrp was a global regulator directly affecting the expression of three positive regulatory genes, and it was a negative regulator involved in the spiramycin and bitespiramycin biosynthesis (Lu et al. 2019). In the biosynthesis regulation of actinomycins, it was found that SCO3361 simultaneously affects the transcription levels of *whiB*, *ssgB*, and *amfC* although SCO3361 only bound with *amfC* (Liu et al. 2017b).

Moreover, by measuring transcription levels of *tobT*, TobR inhibits the transcription of *tobT* through indirect regulation. The transporter TobT may play a very important role in the biosynthesis of tobramycin, and TobR may be involved in the efflux of products or substrate uptake during the secondary metabolism of tobramycin. An increase in TobT transcription level can promote the biosynthesis of tobramycin. And protein TobO was identified as an α-ketoglutarate-dependent non-haem iron amino acid oxidase in *S. tenebrarius* with bioinformatics analysis. Members of the α-ketoglutarate-dependent non-haem iron enzyme superfamily are widely found in prokaryotes, eukaryotes, and archaea. These enzymes typically require Fe^2+^ as a metal cofactor and α-ketoglutarate as a co-substrate for catalysis of various reactions such as hydroxylation, ring cleavage, C–C bond cleavage, cis–trans isomerization, desaturation, intramolecular peroxidation, and heterocycle formation (Gao et al. 2018). Overexpression of *tobO* promoted the biosynthesis of carbamoyltobramycin, and no synthesis blockade or inhibition phenomenon was observed in the knockout strain. Protein function analysis suggested that the catalytic reaction of TobO was not involved in the biosynthetic pathway of carbamoyltobramycin. TobO does not directly provide precursor substances for biosynthesis of carbamoyltobramycin or modify secondary metabolites and intermediate products. The α-ketoglutarate were produced through transamination reactions. Subsequently, under the catalytic action of α-ketoglutarate-dependent non-haem iron enzymes, succinate is generated from α-ketoglutarate and enters central metabolism (Hu et al. 2022; Zhang et al. 2022b). The overexpression of TobR may enhance the decomposition and utilization of amino acids, thereby providing more energy and material for central metabolism through the tricarboxylic acid cycle. Under conditions of higher material and energy utilization efficiency, TobO might promote the growth of mycelium at the primary metabolic stage and provide more producer for the production of secondary metabolite when entering the product synthesis stage. Additionally, adjusting the metabolic flux leads to a reduction in glucose metabolism while increasing the use of amino acids in the system. More glucose is directed towards secondary metabolic synthesis pathways such as carbamoyltobramycin biosynthesis, which requires sufficient precursor materials. As a result, the overexpression of TobO promotes the bacterial growth and the biosynthesis of secondary metabolite.

Finally, the combination strain with *tobR* knockout and *tobO* overexpression has a beneficially additive impact on the production of carbamoyltobramycin, increased by 42.42% (3.76 g/L) compared with strain Tb-*△aprJ* (2.64 g/L). The engineered strains obtained in this study are potentially useful in producing carbamoyltobramycin in the industrial field. In addition, the exploration of novel transcriptional regulatory factors TobR and oxidase TobO in this article can also provide new research ideas for the efficient production of other secondary metabolites.

## Supplementary Information

Below is the link to the electronic supplementary material.

Supplementary file1 (PDF 1139 KB)

## Data Availability

The authors declare that all the data supporting the findings of this study are available within the paper, and its Supplementary Information is available from the corresponding author on request.

## References

[CR1] Bai CX, Zhang Y, Zhao XJ, Hu YL, Xiang SH, Miao J, Lou CB, Zhang LX (2015) Exploiting a precise design of universal synthetic modular regulatory elements to unlock the microbial natural products in. P Natl Acad Sci USA 112(39):12181–12186. 10.1073/pnas.1511027112 10.1073/pnas.1511027112 PMC4593075 26374838

[CR2] Barends DM, Brouwers JCAM, Hulshoff A (1987) Fast precolumn derivatization of aminoglycosides with 1-fluoro-2,4-dinitrobenzene and its application to pharmaceutical analysis. J Pharmaceut Biomed 5(6):613–617. 10.1016/0731-7085(87)80073-0 10.1016/0731-7085(87)80073-0 16867485

[CR3] Bibb MJ, White J, Ward JM, Janssen GR (1994) The messenger-rna for the 23s ribosomal-rna methylase encoded by the erme gene of *Saccharopolyspora-Erythraea* is translated in the absence of a conventional ribosome-binding site. Mol Microbiol 14(3):533–545. 10.1111/j.1365-2958.1994.tb02187.x 7533884 10.1111/j.1365-2958.1994.tb02187.x

[CR4] Brinkman AB, Dahlke I, Tuininga JE, Lammers T, Dumay V, de Heus E, Lebbink JHG, Thomm M, de Vos WM, van der Oost J (2000) An Lrp-like transcriptional regulator from the archaeon *Pyrococcus furiosus* is negatively autoregulated. J Biol Chem 275(49):38160–38169. 10.1074/jbc.M005916200 10973967 10.1074/jbc.M005916200

[CR5] Brinkman AB, Ettema TJG, de Vos WM, van der Oost J (2003) The Lrp family of transcriptional regulators. Mol Microbiol 48(2):287–294. 10.1046/j.1365-2958.2003.03442.x 12675791 10.1046/j.1365-2958.2003.03442.x

[CR6] Chen CW, Huang CH, Lee HH, Tsai HH, Kirby R (2002) Once the circle has been broken: dynamics and evolution of *Streptomyces chromosomes*. Trends Genet 18(10):522–529. 10.1016/S0168-9525(02)02752-X 12350342 10.1016/s0168-9525(02)02752-x

[CR7] Chen YH, Smanski MJ, Shen B (2010) Improvement of secondary metabolite production in *Streptomyces* by manipulating pathway regulation. Appl Microbiol Biot 86(1):19–25. 10.1007/s00253-009-2428-3 10.1007/s00253-009-2428-3 PMC3472513 20091304

[CR8] de los Rios S, Perona JJ (2007) Structure of the Escherichia coli leucine-responsive regulatory protein Lrp reveals a novel octameric assembly. J Mol Biol 366(5):1589–1602. 10.1016/j.jmb.2006.12.032 17223133 10.1016/j.jmb.2006.12.032 PMC1933502

[CR9] Dong HJ, Yue X, Yan BY, Gao W, Wang S, Li YQ (2020) Improved A40926 production from using the promoter engineering and the co-expression of crucial genes. J Biotechnol 324:28–33. 10.1016/j.jbiotec.2020.09.017 32971181 10.1016/j.jbiotec.2020.09.017

[CR10] Ettema TJG, Brinkman AB, Tani TH, Rafferty JB, van der Oost J (2002) A novel ligand-binding domain involved in regulation of amino acid metabolism in prokaryotes. J Biol Chem 277(40):37464–37468. 10.1074/jbc.M206063200 12138170 10.1074/jbc.M206063200

[CR11] Fan PH, Sato S, Yeh YC, Liu HW (2023) Biosynthetic Origin of the octose core and its mechanism of assembly during apramycin biosynthesis. J Am Chem Soc 145(39):21361–21369. 10.1021/jacs.3c06354 37733880 10.1021/jacs.3c06354 PMC10591738

[CR12] Gao SS, Naowarojna N, Cheng RH, Liu XT, Liu PH (2018) Recent examples of -ketoglutarate-dependent mononuclear non-haem iron enzymes in natural product biosyntheses. Nat Prod Rep 35(8):792–837. 10.1039/c7np00067g 29932179 10.1039/c7np00067g PMC6093783

[CR13] Hellman LM, Fried MG (2007) Electrophoretic mobility shift assay (EMSA) for detecting protein-nucleic acid interactions. Nat Protoc 2(8):1849–1861. 10.1038/nprot.2007.249 17703195 10.1038/nprot.2007.249 PMC2757439

[CR14] Hong W, Yan S (2012) Engineering *Streptomyces tenebrarius* to synthesize single component of carbamoyltobramycin. Lett Appl Microbiol 55(1):33–39. 10.1111/j.1472-765X.2012.03254.x 22509935 10.1111/j.1472-765X.2012.03254.x

[CR15] Hu SW, Li YY, Zhang AL, Li H, Chen KQ, Ouyang PK (2022) Designing of an efficient whole-cell biocatalyst system for converting l-lysine into cis-3-hydroxypipecolic acid. Front Microb 13:945184. 10.3389/fmicb.2022.945184. (**ARTN**) 10.3389/fmicb.2022.945184 PMC9271919 35832817

[CR16] Jeong JA, Hyun J, Oh JI (2015) Regulation mechanism of the ald gene encoding alanine dehydrogenase in *Mycobacterium smegmatis* and *Mycobacterium tuberculosis* by the Lrp/AsnC family regulator AldR. J Bacteriol 197(19):3142–3153. 10.1128/Jb.00453-15 26195594 10.1128/JB.00453-15 PMC4560286

[CR17] Kawashima T, Aramaki H, Oyamada T, Makino K, Yamada M, Okamura H, Yokoyama K, Ishijima SA, Suzuki M (2008) Transcription regulation by feast/famine regulatory proteins, FFRPs in archaea and eubacteria. Biol Pharm Bull 31(2):173–186. 10.1248/bpb.31.173 18239270 10.1248/bpb.31.173

[CR18] Kharel MK, Basnet DB, Lee HC, Liou K, Woo JS, Kim BG, Sohng JK (2004) Isolation and characterization of the tobramycin biosynthetic gene cluster from *Streptomyces tenebrarius*. FEMS Microbiol Lett 230(2):185–190. 10.1016/S0378-1097(03)00881-4 14757238 10.1016/S0378-1097(03)00881-4

[CR19] Kim HJ, LeVieux J, Yeh YC, Liu HW (2016) C3-Deoxygenation of paromamine catalyzed by a radical s-adenosylmethionine enzyme: characterization of the enzyme AprD4 and its reductase partner AprD3. Angew Chem Int Edit 55(11):3724–3728. 10.1002/anie.201510635 10.1002/anie.201510635 PMC4943880 26879038

[CR20] Koch KF, Davis FA, Rhoades JA (1973) Nebramycin: separation of the complex and identification of factors 4, 5, and 5’. J Antibiot (tokyo) 26(12):745–751. 10.7164/antibiotics.26.745 4792386 10.7164/antibiotics.26.745

[CR21] Koike H, Ishijima SA, Clowney L, Suzuki M (2004) The archaeal feast/famine regulatory protein: potential roles of its assembly forms for regulating transcription. P Natl Acad Sci USA 101(9):2840–2845. 10.1073/pnas.0400109101 10.1073/pnas.0400109101 PMC365707 14976242

[CR22] Kudo F, Eguchi T (2009) Biosynthetic genes for aminoglycoside antibiotics. J Antibiot (tokyo) 62(9):471–481. 10.1038/ja.2009.76 19644520 10.1038/ja.2009.76

[CR23] Kudo F, Tokumitsu T, Eguchi T (2017) Substrate specificity of radical S-adenosyl-l-methionine dehydratase AprD4 and its partner reductase AprD3 in the C3’-deoxygenation of aminoglycoside antibiotics. J Antibiot (tokyo) 70(4):423–428. 10.1038/ja.2016.110 10.1038/ja.2016.110 27599765

[CR24] Kudo F, Mori A, Koide M, Yajima R, Takeishi R, Miyanaga A, Eguchi T (2021) One-pot enzymatic synthesis of 2-deoxy-scyllo-inosose from d-glucose and polyphosphate. Biosci Biotech Bioch 85(1):108–114. 10.1093/bbb/zbaa025 10.1093/bbb/zbaa025 33577648

[CR25] Leonard PM, Smits SHJ, Sedelnikova SE, Brinkman AB, de Vos WM, van der Oost J, Rice DW, Rafferty JB (2001) Crystal structure of the Lrp-like transcriptional regulator from the archaeon *Pyrococcus furiosus*. Embo J 20(5):990–997. 10.1093/emboj/20.5.990 11230123 10.1093/emboj/20.5.990 PMC145483

[CR26] Liu J, Chen YF, Wang WW, Ren M, Wu PP, Wang YS, Li CR, Zhang LX, Wu H, Weaver DT, Zhang BC (2017a) Engineering of an Lrp family regulator SACE_Lrp improves erythromycin production in *Saccharopolyspora erythraea*. Metab Eng 39:29–37. 10.1016/j.ymben.2016.10.012 27794466 10.1016/j.ymben.2016.10.012

[CR27] Liu J, Li J, Dong H, Chen YF, Wang YS, Wu H, Li CR, Weaver DT, Zhang LX, Zhang BC (2017b) Characterization of an Lrp/AsnC family regulator SCO3361, controlling actinorhodin production and morphological development in *Streptomyces coelicolor*. Appl Microbiol Biot 101(14):5773–5783. 10.1007/s00253-017-8339-9 10.1007/s00253-017-8339-9 28601893

[CR28] Liu J, Chen YF, Li L, Yang ED, Wang YS, Wu H, Zhang LX, Wang WY, Zhang BC (2019) Characterization and engineering of the Lrp/AsnC family regulator SACE_5717 for erythromycin overproduction in *Saccharopolyspora erythraea*. J Ind Microbiol Biot 46(7):1013–1024. 10.1007/s10295-019-02178-2 10.1007/s10295-019-02178-2 31016583

[CR29] Liu J, Li L, Wang YX, Li BW, Cai XL, Tang LJ, Dong SN, Yang ED, Wu H, Zhang BC (2021) Joint engineering of SACE_Lrp and its target MarR enhances the biosynthesis and export of erythromycin in *Saccharopolyspora erythraea*. Appl Microbiol Biot 105(7):2911–2924. 10.1007/s00253-021-11228-8 10.1007/s00253-021-11228-8 33760930

[CR30] Liu J, Wang YX, He HY, Dong SN, Tang LJ, Yang ED, Wang WY, Zhang BC (2023) The leucine-responsive regulatory protein SCAB_Lrp modulates thaxtomin biosynthesis, pathogenicity, and morphological development in *Streptomyces*. Mol Plant Pathol 24(2):167–178. 10.1111/mpp.13285 36478143 10.1111/mpp.13285 PMC9831280

[CR31] Lu ZL, Zhang XT, Dai JL, Wang YG, He WQ (2019) Engineering of leucine-responsive regulatory protein improves spiramycin and bitespiramycin biosynthesis. Microb Cell Fact 18:38. 10.1186/s12934-019-1086-0. (**ARTN**) 30782164 10.1186/s12934-019-1086-0 PMC6379999

[CR32] Lv M, Ji X, Zhao J, Li Y, Zhang C, Su L, Ding W, Deng Z, Yu Y, Zhang Q (2016) Characterization of a C3 deoxygenation pathway reveals a key branch point in aminoglycoside biosynthesis. J Am Chem Soc 138(20):6427–6435. 10.1021/jacs.6b02221 27120352 10.1021/jacs.6b02221

[CR33] Macneil DJ, Gewain KM, Ruby CL, Dezeny G, Gibbons PH, Macneil T (1992) Analysis of *Streptomyces-Avermitilis* genes required for avermectin biosynthesis utilizing a novel integration vector. Gene 111(1):61–68. 10.1016/0378-1119(92)90603-M 1547955 10.1016/0378-1119(92)90603-m

[CR34] Mitousis L, Maier H, Martinovic L, Kulik A, Stockert S, Wohlleben W, Stiefel A, Musiol-Kroll EM (2021) Engineering of *Streptoalloteichus tenebrarius* 2444 for sustainable production of tobramycin. Molecules 26(14). 10.3390/molecules26144343 10.3390/molecules26144343 PMC8304502 34299618

[CR35] Myronovskyi M, Luzhetskyy A (2016) Native and engineered promoters in natural product discovery. Nat Prod Rep 33(8):1006–1019. 10.1039/c6np00002a 27438486 10.1039/c6np00002a

[CR36] Ni X, Li D, Yang L, Huang T, Li H, Xia H (2011) Construction of kanamycin B overproducing strain by genetic engineering of *Streptomyces tenebrarius*. Appl Microbiol Biotechnol 89(3):723–731. 10.1007/s00253-010-2908-5 20936279 10.1007/s00253-010-2908-5

[CR37] Oconnor S, Lam LKT, Jones ND, Chaney MO (1976) Apramycin, a unique aminocyclitol antibiotic. J Org Chem 41(12):2087–2092. 10.1021/jo00874a003 932851 10.1021/jo00874a003

[CR38] Pagkalis S, Mantadakis E, Mavros MN, Ammari C, Falagas ME (2011) Pharmacological considerations for the proper clinical use of aminoglycosides. Drugs 71(17):2277–2294. 10.2165/11597020-000000000-00000 22085385 10.2165/11597020-000000000-00000

[CR39] Park JW, Park SR, Nepal KK, Han AR, Ban YH, Yoo YJ, Kim EJ, Kim EM, Kim D, Sohng JK, Yoon YJ (2011) Discovery of parallel pathways of kanamycin biosynthesis allows antibiotic manipulation. Nat Chem Biol 7(11):843–852. 10.1038/nchembio.671 21983602 10.1038/nchembio.671

[CR40] Park SR, Park JW, Ban YH, Sohng JK, Yoon YJ (2013) 2-Deoxystreptamine-containing aminoglycoside antibiotics: recent advances in the characterization and manipulation of their biosynthetic pathways. Nat Prod Rep 30(1):11–20. 10.1039/c2np20092a 23179168 10.1039/c2np20092a

[CR41] Parthier C, Gorlich S, Jaenecke F, Breithaupt C, Brauer U, Fandrich U, Clausnitzer D, Wehmeier UF, Bottcher C, Scheel D, Stubbs MT (2012) The O-carbamoyltransferase TobZ catalyzes an ancient enzymatic reaction. Angew Chem Int Ed Engl 51(17):4046–4052. 10.1002/anie.201108896 22383337 10.1002/anie.201108896

[CR42] Peeters E, Charlier D (2010) The Lrp family of transcription regulators in *Archaea*. Archaea 2010:750457. 10.1155/2010/750457. (**Artn**) 21151646 10.1155/2010/750457 PMC2995911

[CR43] Pritchett MA, Wilkinson SP, Geiduschek EP, Ouhammouch M (2009) Hybrid Ptr2-like activators of archaeal transcription. Mol Microbiol 74(3):582–593. 10.1111/j.1365-2958.2009.06884.x 19775246 10.1111/j.1365-2958.2009.06884.x

[CR44] Qattan SYA, Khattab AA (2019) Molecular characterization of *Streptomyces albogriseolus* excellent mutants for neomycin production. J Pure Appl Microbio 13(3):1489–1498. 10.22207/Jpam.13.3.20. (**Artn 5626**)

[CR45] Reddy MCM, Gokulan K, Jacobs WR, Ioerger TR, Sacchettini JC (2008) Crystal structure of Mycobacterium tuberculosis LrpA, a leucine-responsive global regulator associated with starvation response. Protein Sci 17(1):159–170. 10.1110/ps.073192208 18042675 10.1110/ps.073192208 PMC2144582

[CR46] Rosalia M, Chiesa E, Tottoli EM, Dorati R, Genta I, Conti B, Pisani S (2022) Tobramycin nanoantibiotics and their advantages: a minireview. Int J Mol Sci 23(22):14080. 10.3390/ijms232214080. (**ARTN**) 36430555 10.3390/ijms232214080 PMC9692674

[CR47] Schwaiger R, Schwarz C, Furtwangler K, Tarasov V, Wende A, Oesterhelt D (2010) Transcriptional control by two leucine-responsive regulatory proteins in *Halobacterium salinarum* R1. Bmc Mol Biol 11:40. 10.1186/1471-2199-11-40. (**Artn**) 20509863 10.1186/1471-2199-11-40 PMC2894021

[CR48] Song NN, Duc TN, van Oeffelen L, Muyldermans S, Peeters E, Charlier D (2013) Expanded target and cofactor repertoire for the transcriptional activator LysM from *Sulfolobus*. Nucleic Acids Res 41(5):2932–2949. 10.1093/nar/gkt021 23355617 10.1093/nar/gkt021 PMC3597687

[CR49] Strieker M, Kopp F, Mahlert C, Essen LO, Marahiel MA (2007) Mechanistic and structural basis of stereospecific Cbeta-hydroxylation in calcium-dependent antibiotic, a daptomycin-type lipopeptide. ACS Chem Biol 2(3):187–196. 10.1021/cb700012y 17373765 10.1021/cb700012y

[CR50] Sun JH, Kelemen GH, Fernández-Abalos JM, Bibb MJ (1999) Green fluorescent protein as a reporter for spatial and temporal gene expression in A3(2). Microbiol-Uk 145:2221–2227. 10.1099/00221287-145-9-2221 10.1099/00221287-145-9-2221 10517575

[CR51] Sun JY, Gao HJ, Yan DY, Liu Y, Ni XP, Xia HZ (2022) Characterization and utilization of methyltransferase for apramycin production in *Streptoalloteichus tenebrarius*. J Ind Microbiol Biot 49(4) 10.1093/jimb/kuac011 10.1093/jimb/kuac011 PMC9338882 35536571

[CR52] Tamegai H, Eguchi T, Kakinuma K (2002) First identification of Streptomyces genes involved in the biosynthesis of 2-deoxystreptamine-containing aminoglycoside antibiotics-genetic and evolutionary analysis of L-glutamine:2-deoxy-scyllo-inosose aminotransferase genes. J Antibiot (tokyo) 55(11):1016–1018. 10.7164/antibiotics.55.1016 12546424 10.7164/antibiotics.55.1016

[CR53] Tamura T, Ishida Y, Otoguro M, Hatano K, Suzuki K (2008) Classification of '*Streptomyces tenebrarius*’ Higgins and Kastner as *Streptoalloteichus tenebrarius* nom. rev., comb. nov., and emended description of the genus *Streptoalloteichus*. Int J Syst Evol Microbiol 58(Pt 3):688–91. 10.1099/ijs.0.65272-0 18319479 10.1099/ijs.0.65272-0

[CR54] Wang L, Pulk A, Wasserman MR, Feldman MB, Altman RB, Cate JH, Blanchard SC (2012) Allosteric control of the ribosome by small-molecule antibiotics. Nat Struct Mol Biol 19(9):957–963. 10.1038/nsmb.2360 22902368 10.1038/nsmb.2360 PMC3645490

[CR55] Wang W, Li X, Wang J, Xiang S, Feng X, Yang K (2013) An engineered strong promoter for streptomycetes. Appl Environ Microbiol 79(14):4484–4492. 10.1128/AEM.00985-13 23686264 10.1128/AEM.00985-13 PMC3697493

[CR56] Wang JX, Ma SZ, Ding W, Chen T, Zhang Q (2021) Mechanistic study of oxidoreductase AprQ involved in biosynthesis of aminoglycoside antibiotic apramycin. Chin J Chem 39(7):1923–1926. 10.1002/cjoc.202100070

[CR57] Wasserman MR, Pulk A, Zhou Z, Altman RB, Zinder JC, Green KD, Garneau-Tsodikova S, Cate JHD, Blanchard SC (2015) Chemically related 4,5-linked aminoglycoside antibiotics drive subunit rotation in opposite directions. Nat Commun 6:7896. 10.1038/ncomms8896. (**ARTN**) 26224058 10.1038/ncomms8896 PMC4522699

[CR58] Wehmeier UF, Piepersberg W (2009) Enzymology of aminoglycoside biosynthesis-deduction from gene clusters. Method Enzymol 459:459–491. 10.1016/S0076-6879(09)04619-9 10.1016/S0076-6879(09)04619-9 19362651

[CR59] Xiao J, Li H, Wen S, Hong W (2014) Concentrated biosynthesis of tobramycin by genetically engineered *Streptomyces tenebrarius*. J Gen Appl Microbiol 60(6):256–261. 10.2323/jgam.60.256 25742977 10.2323/jgam.60.256

[CR60] Xu YR, Tang YQ, Wang N, Liu J, Cai XL, Cai HY, Li J, Tan GQ, Liu RH, Bai LQ, Zhang LX, Wu H, Zhang BC (2020) Transcriptional regulation of a leucine-responsive regulatory protein for directly controlling lincomycin biosynthesis in *Streptomyces lincolnensis*. Appl Microbiol Biot 104(6):2575–2587. 10.1007/s00253-020-10381-w 10.1007/s00253-020-10381-w 31993701

[CR61] Xu YR, Xu WL, Yi J, Li BL, Liu M, Zhang MF, Zheng Y, Liu RH, Wu H, Zhang BC (2023) Transcriptomics-guided investigation of the SLCG_Lrp regulon provides new insights into its role for lincomycin biosynthesis. Fermentation-Basel 9(4):396. 10.3390/fermentation9040396. (**ARTN**)

[CR62] Yu LJ, Pan YY, Liu G (2016) A regulatory gene *SCO2140* is involved in antibiotic production and morphological differentiation of *Streptomyces coelicolor* A3(2). Curr Microb 73(2):196–201. 10.1007/s00284-016-1050-8 10.1007/s00284-016-1050-8 27113590

[CR63] Zhang Q, Chi HT, Wu L, Deng Z, Yu Y (2021) Two cryptic self-resistance mechanisms in *Streptomyces tenebrarius* reveal insights into the biosynthesis of apramycin. Angew Chem Int Ed Engl 60(16):8990–8996. 10.1002/anie.202100687 33538390 10.1002/anie.202100687

[CR64] Zhang Q, He C, Sun J, Deng ZX, Yu Y (2022a) 7′ methylation in apramycin: its biosynthesis and biological role. Org Chem Front 9(10):2708–2713. 10.1039/d2qo00260d

[CR65] Zhang Y, Zhao J, Wang XL, Tang Y, Liu SW, Wen TY (2022b) Model-guided metabolic rewiring for gamma-aminobutyric acid and butyrolactam biosynthesis in *Corynebacterium glutamicum* ATCC13032. Biology-Basel 11(6):846. 10.3390/biology11060846. (**ARTN**) 35741367 10.3390/biology11060846 PMC9219837

[CR66] Ziegler CA, Freddolino PL (2021) The leucine-responsive regulatory proteins/feast-famine regulatory proteins: an ancient and complex class of transcriptional regulators in bacteria and archaea. Crit Rev Biochem Mol Biol 56(4):373–400. 10.1080/10409238.2021.1925215 34151666 10.1080/10409238.2021.1925215 PMC9239533

