# Supplementary material for: Improving the production of carbamoyltobramycin by an industrial Streptoalloteichus tenebrarius through metabolic engineering
Source: Appl Microbiol Biotechnol. 2024 Apr 21;108:304. doi: 10.1007/s00253-024-13141-2 (PMC11033246; doi:10.1007/s00253-024-13141-2)
Supplement: Supplementary file 1 [file 253_2024_13141_MOESM1_ESM.pdf]

Applied Microbiology and Biotechnology

Improving the production of carbamoyltobramycin by an industrial *Streptoalloteichus tenebrarius* through metabolic engineering

Yun Feng[1], Yiqi Jiang[1], XuTong Chen[1], Li Zhu[1], Hailong Xue[1], Mianbin Wu[1], Lirong Yang[1, 2], Haoran Yu[1, 2, *], Jianping Lin[1, *]

[1] Key Laboratory of Biomass Chemical Engineering of Ministry of Education, College of Chemical and Biological Engineering, Zhejiang University, Hangzhou, 310058, China

[2] Hangzhou Global Scientific and Technological Innovation Center, Zhejiang University, Hangzhou, 311200, China

[*]For correspondence: Haoran Yu, E-mail: yuhaoran@zju.edu.cn; Jianping Lin, E-mail: linjp@zju.edu.cn

Supplementary Figure

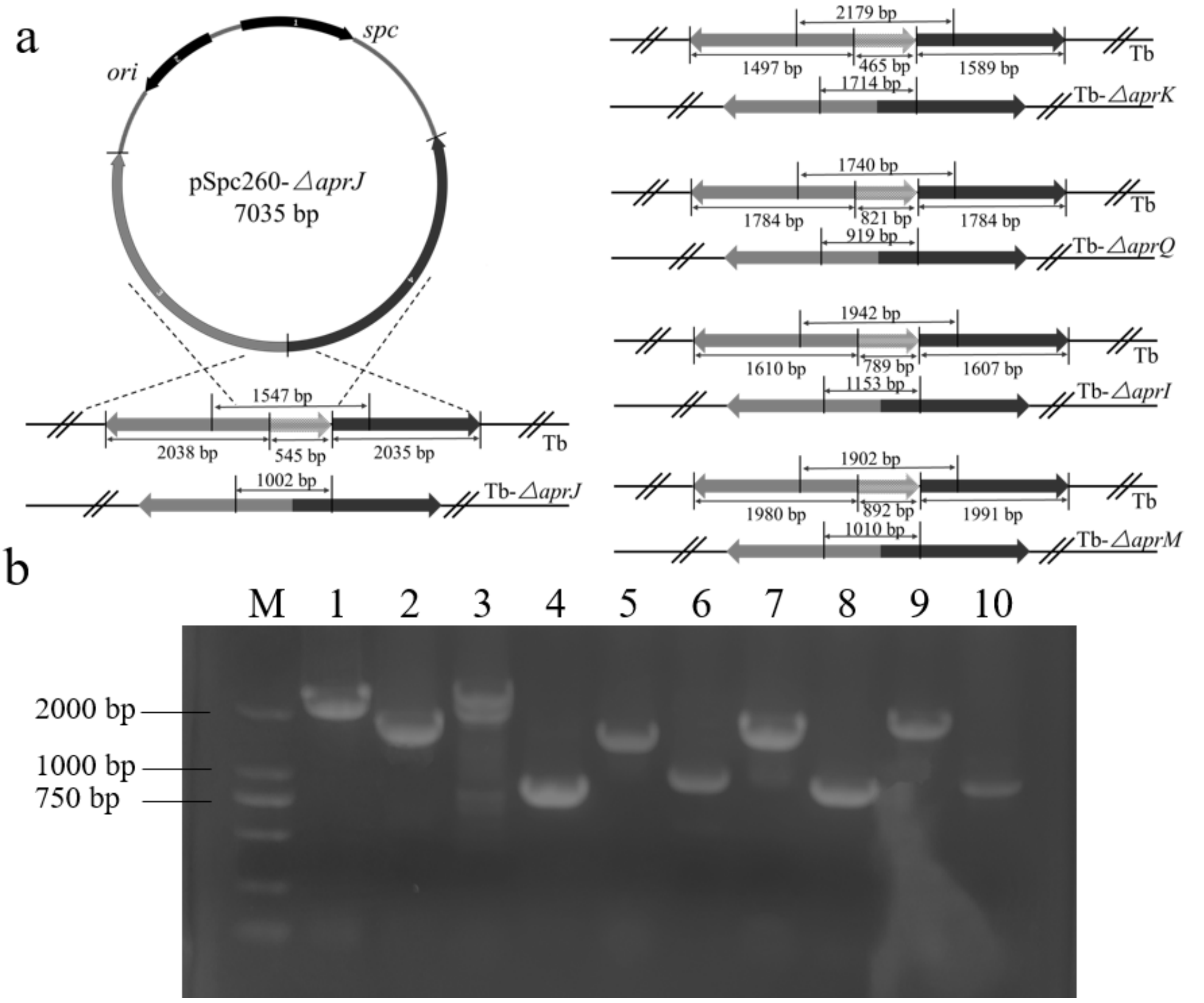

Figure S1. Construction of strains and plasmids for the elimination of byproduct apramycin. a Construction of inactivation plasmid pSpc260-*△aprJ* and the genotype of mutants Tb-*△aprJ*, Tb-*△aprK*, Tb-*△aprQ*, Tb-*△aprI* and Tb-*△aprM*. **b** Identification of Tb-*△aprJ*, Tb-*△aprK*, Tb-*△aprQ*, Tb-*△aprI* and Tb-*△aprM* by PCR. The template of lane 1 was from Tb genomes with a 2179 bp band, the template of lane 2 was from *△aprK* mutant genomes with a 1714 bp band, primers di-aprK-F and di-aprK-R were used in this part, consistent with the expected deletion of 465 bp fragment within the region of the *aprK*. The template of lane 3 was from Tb genomes with a 1942 bp band, the template of lane 4 was from *△aprI* mutant genomes with a 1153 bp band, primers di-aprI-F and di-aprI-R were used in this part, consistent with the expected deletion of 789 bp fragment within the region of the *aprI*. The template of lane 5 was from Tb genomes with a 1547 bp band, the template of lane 6 was from *△aprJ* mutant genomes with a 1002 bp band, primers di-aprJ-F and di-aprJ-R were used in this part, consistent with the expected deletion of 545 bp fragment within the region of the *aprJ*. The template of lane 7 was from Tb genomes with a 1740 bp band, the template of lane 8 was from *△aprQ* mutant genomes with a 910 bp band, primers di-aprQ-F and di-aprQ-R were used in this part, consistent with the expected deletion of 821 bp fragment within the region of the *aprQ*. The template of lane 9 was from

Tb genomes with a 1902 bp band, the template of lane 10 was from *△aprM* mutant genomes with a 1010 bp band, primers di-aprM-F and di-aprM-R were used in this part, consistent with the expected deletion of 892 bp fragment within the region of the *aprM*.

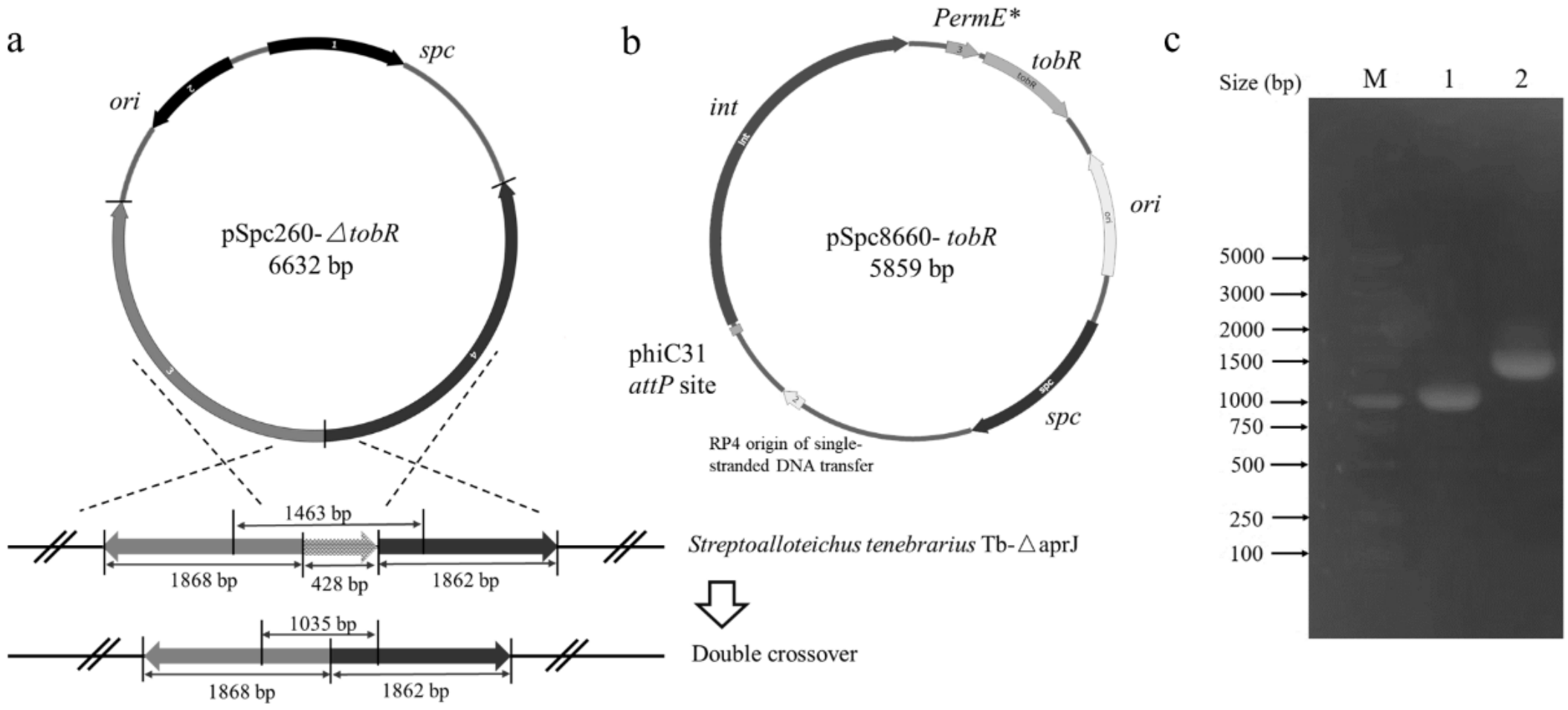

Figure S2. Construction of strains and plasmids for TobR research. a Construction of inactivation plasmid pSpc260-*△tobR*. **b** Construction of overexpression plasmid pSpc8660-*tobR*. **c** Identification of *△tobR* mutant by PCR. The template of lane 1 was from Tb-*△aprJ*-*△tobR* mutant genomes with a 1035 bp band and template of lane 2 was from Tb-*△aprJ* genomes with a 1463 bp band, consistent with the expected deletion of 428 bp fragment within the region of the *tobR*.

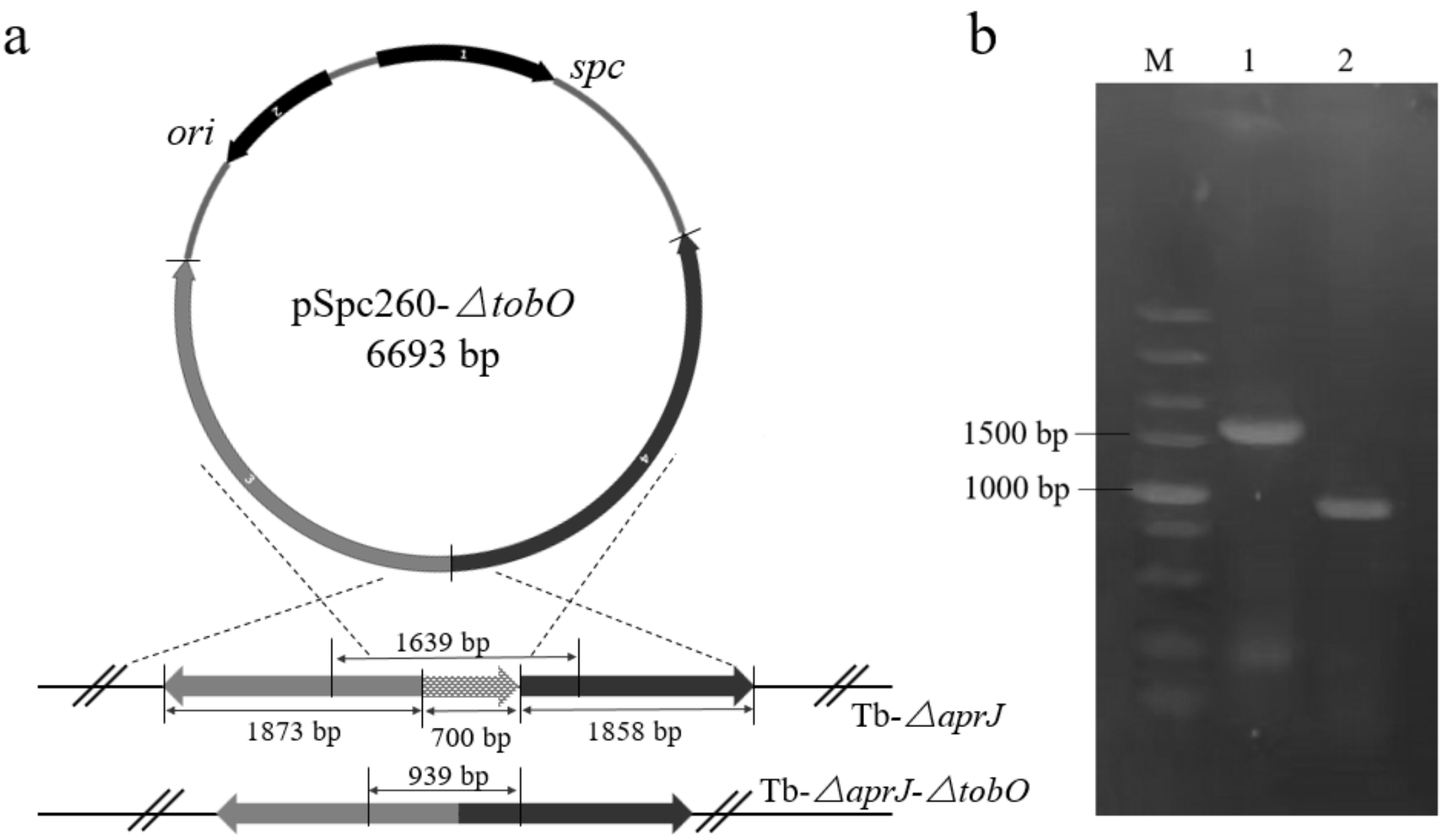

Figure S3. Construction of strain Tb-*△aprJ*-*△tobO*. a Construction of inactivation plasmid pSpc260-*△tobO*. **b** Identification of *△tobO* mutant by PCR. The template of lane 1 was from Tb-*△aprJ* genomes with a 1639 bp band, the

template of lane 2 was from Tb-*△aprJ*-*△tobO* mutant genomes with a 939 bp band, primers di-tobO-F and di-tobO-R were used in this part, consistent with the expected deletion of 700 bp fragment within the region of the *tobO*.

glycosyltransferase family 4 protein [Streptoalloteichus tenebrarius]

Sequence ID: WP_253672175.1 Length: 439 Number of Matches: 1

See 2 more title(s) See all Identical Proteins(IPG)

Range 1: 1 to 439 GenPept Graphics ▼ Next Match ▲ Previous Match

Score	Expect	Method	Identities	Positives	Gaps
857 bits(2213)	0.0	Compositional matrix adjust.	438/439(99%)	439/439(100%)	0/439(0%)

```
Query  1    VRVLRLTPFFHHDCVDSWPAEFDSVGGMQVQILRLSRQLAQRGVRQEVFTVGFPGLPRVR  60
            +RVLRLTPFFHHDCVDSWPAEFDSVGGMQVQILRLSRQLAQRGVRQEVFTVGFPGLPRVR
Sbjct  1    MRVLRLTPFFHHDCVDSWPAEFDSVGGMQVQILRLSRQLAQRGVRQEVFTVGFPGLPRVR  60

Query  61   EDSPGLVVRITRAPMPRLRSELTGLVGLNLAWFLGAMAECLRRRRGPLPDLIQVHGDGQL  120
            EDSPGLVVRITRAPMPRLRSELTGLVGLNLAWFLGAMAECLRRRRGPLPDLIQVHGDGQL
Sbjct  61   EDSPGLVVRITRAPMPRLRSELTGLVGLNLAWFLGAMAECLRRRRGPLPDLIQVHGDGQL  120

Query  121  WALLAGPLASAILRRPYSLVLHCSRLGVYQPMSRYDRWQHRFVAAVERWAVRRASGVCAL  180
            WALLAGPLASAILRRPYSLVLHCSRLGVYQPMSRYDRWQHRFVAAVERWAVRRASGVCAL
Sbjct  121  WALLAGPLASAILRRPYSLVLHCSRLGVYQPMSRYDRWQHRFVAAVERWAVRRASGVCAL  180

Query  181  TTRTADVVRKALRPHQVRVDVVPDSVDPDPPAHSGVPVADRLRAAGLPPDARVVGYVGRV  240
            TTRTADVVRKALRPHQVRVDVVPDSVDPDPPAHSGVPVADRLRAAGLPPDARVVGYVGRV
Sbjct  181  TTRTADVVRKALRPHQVRVDVVPDSVDPDPPAHSGVPVADRLRAAGLPPDARVVGYVGRV  240

Query  241  AHEKGWSHFVDVAERLAGGPAGERVVFLVVGDGPQRPRMAERVAAAGLADRFVFTGFLPN  300
            AHEKGWSHFVDVAERLAGGPAGERVVFLVVGDGPQRPRMAERVAAAGLADRFVFTGFLPN
Sbjct  241  AHEKGWSHFVDVAERLAGGPAGERVVFLVVGDGPQRPRMAERVAAAGLADRFVFTGFLPN  300

Query  301  QDIPLTMGGIDVLVMPSVHEELGGSAIEAMVLGVPVVAYGVGGLRDTVGRVTPSLAVRPQ  360
            QDIPLTMGGIDVLVMPSVHEELGGSAIEAMVLGVPVVAYGVGGLRDTVGRVTPSLAVRPQ
Sbjct  301  QDIPLTMGGIDVLVMPSVHEELGGSAIEAMVLGVPVVAYGVGGLRDTVGRVTPSLAVRPQ  360

Query  361  DVGALTDAVRDVLARTDEYRAQVRAGRPWLEENYGDGVGVTRTVAHYHRILAGGRGGVTA  420
            DVGALTDAVRDVLARTDEYRAQVRAGRPWLEENYGDGVGVTRTVAHYHRILAGGRGGVTA
Sbjct  361  DVGALTDAVRDVLARTDEYRAQVRAGRPWLEENYGDGVGVTRTVAHYHRILAGGRGGVTA  420

Query  421  GAGLPDDPARAGRSPASGR  439
            GAGLPDDPARAGRSPASGR
Sbjct  421  GAGLPDDPARAGRSPASGR  439
```

Figure S4. Functional analysis of AprM through NCBI database

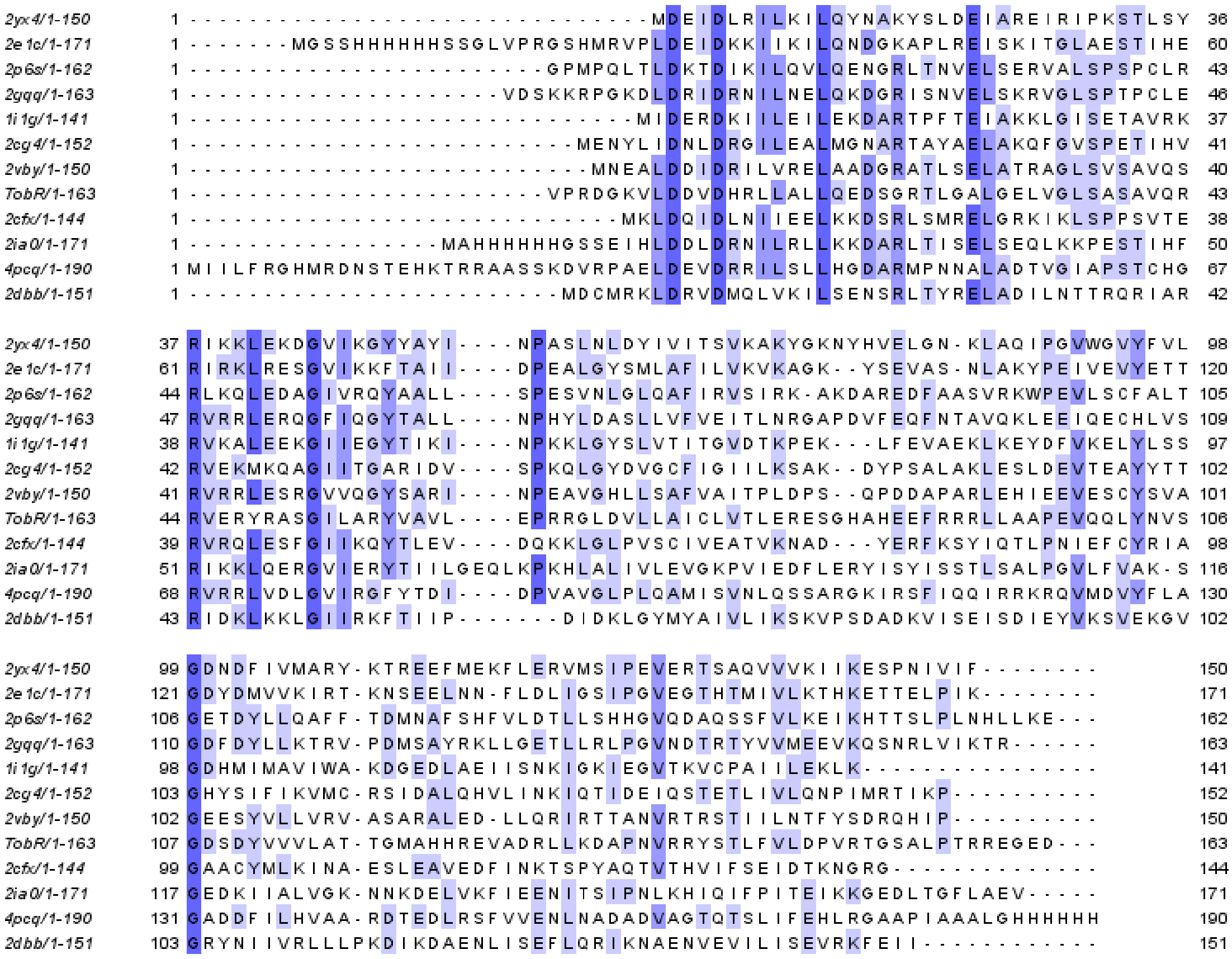

Figure S5. The amino acid sequences of TobR and the other Lrp/AsnC family transcription regulars. All sequences (PDB ID: 4PCQ, 2CFX, 2VBY, 2GQQ, 2P6S, 2DBB, 2IA0, 2CG4, 1I1G, 2E1C and 2YX4) were aligned by CLUSTALW. Blue depth indicates the percentage identity of conservation.

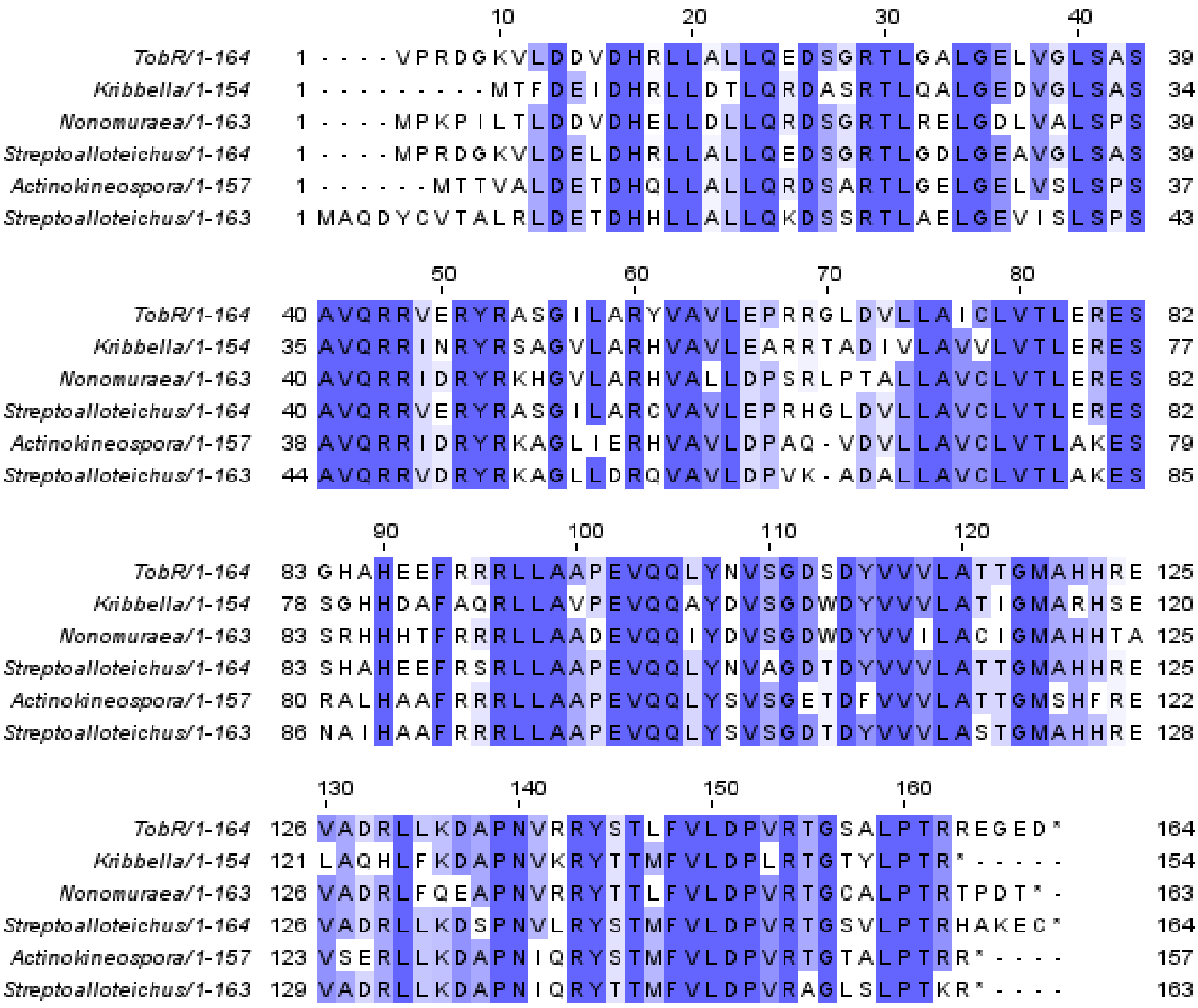

Figure S6. The amino acid sequences of TobR and its homologous proteins. The homologous proteins were from *Streptoalloteichus hindustanus* (WP_073480789.1), *Actinokineospora alba* (WP_228769743.1), *Alloactinosynnema sp.* L-07 (CRK55752.1), *Nonomuraea sp.* KC401 (WP_138203008.1) and *Kribbella antibiotica* (WP_138203008.1) are 68.89%-88.27% similar. The alignment was analyzed by Clustal Omega. Blue depth indicates the percentage identity of conservation.

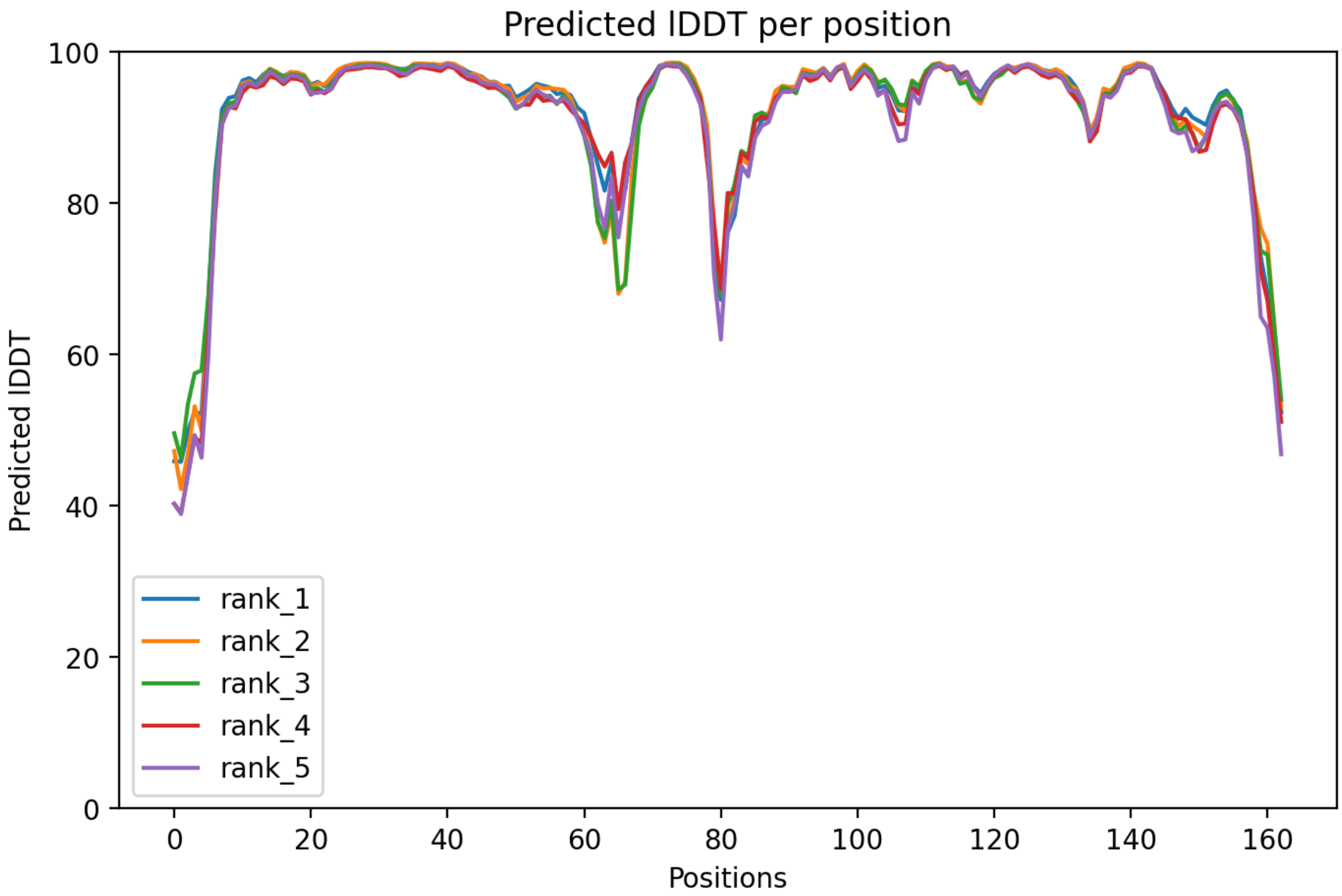

Figure S7. plDDT score for the TobR structure predicted by Alphafold 2.

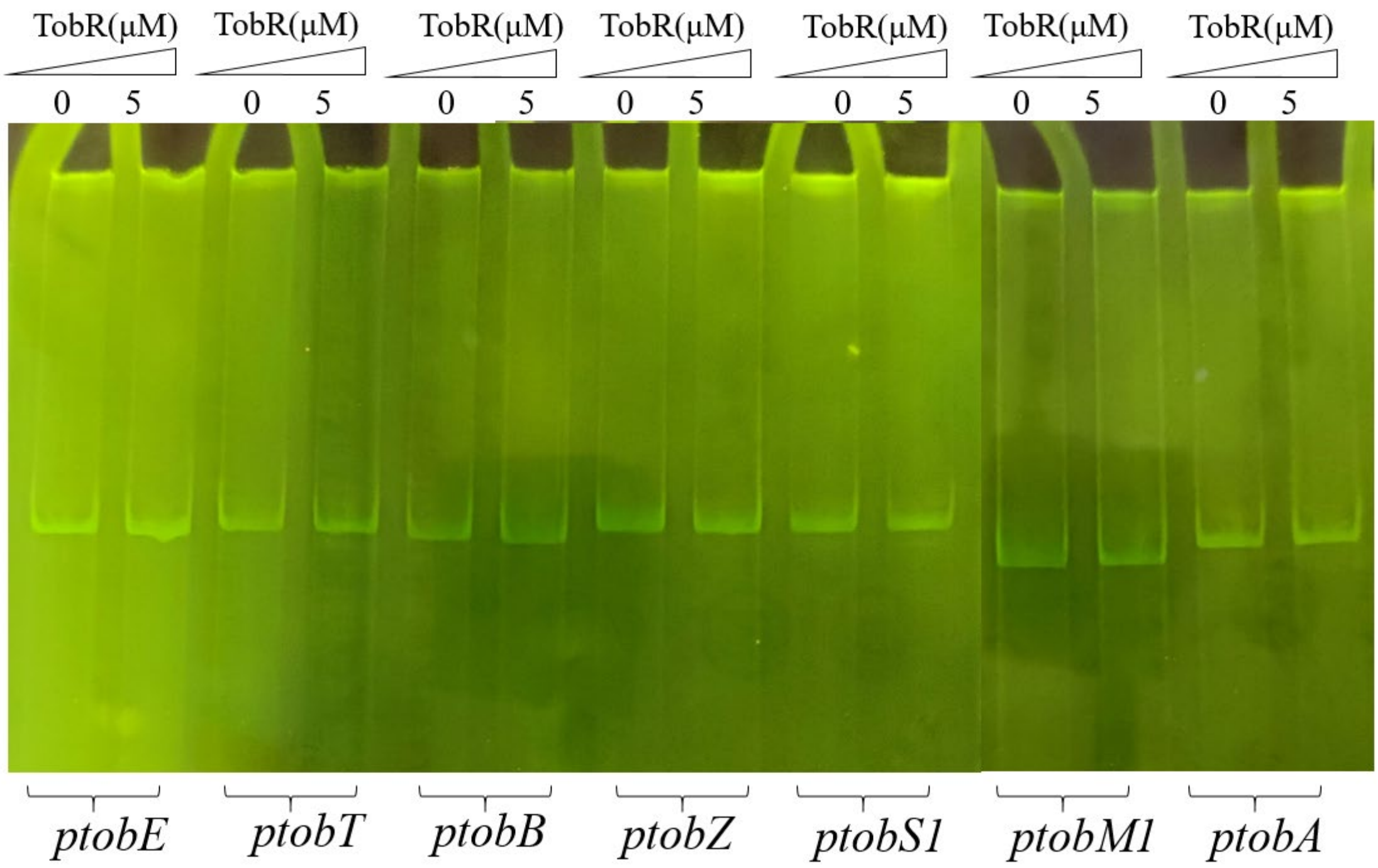

Figure S8. The regulatory targets of TobR on tobramycin biosynthesis gene cluster analyzed by EMSA. The protein concentrations in the experimental groups were 5 μM.

Supplementary Tables

Table S1 Plasmids used in this study.

Plasmids	Relevant characteristic	Source
pOJ260	*E. coli*, cloning vector, containing pUC18 replicon, *oriT*, *aac3(IV)*	Changsha Yingrun Biotechnology Co.,Ltd (Hunan, China)
pSpc260	*E. coli*, cloning vector, containing pUC18 replicon, *oriT*, *spc*, derived from pOJ260	This study
pSpc260-*△aprJ*	pSpc260 deritative for *aprJ* deletion	This study
pSpc260-*△aprK*	pSpc260 deritative for *aprK* deletion	This study
pSpc260-*△aprI*	pSpc260 deritative for *aprI* deletion	This study
pSpc260-*△aprQ*	pSpc260 deritative for *aprQ* deletion	This study
pSpc260-*△aprM*	pSpc260 deritative for *aprM* deletion	This study
pSpc260-*△tobR*	pSpc260 deritative for *tobR* deletion	This study
pSpc260-*△tobO*	pSpc260 deritative for *tobO* deletion	This study
pIJ8660	*aac3(IV)*	(Sun et al. 1999)
pIJ8660-*spc*	*spc*, originl, derived from pIJ8660	This study
pSpc8660	*spc*, *ermEp** originl, derived from pIJ8660-*spc*	This study
pSpc8660-*tobR*	pSpc8660 derivative for expression of *tobR*	This study
pSpc8660-*tobO*	pSpc8660 derivative for expression of *tobO*	This study
pSpc-*kasOp**	*spc*, *kasOp** originl, derived from pSpc8660	This study
pSpc-*kasOp*-tobO*	pSpc-*kasOp** derivative for expression of *tobO*	This study
pET28a(+)	*kan*, P_{T7}, His-tag, vector for protein expression	Sangon Biotech (Shanghai, China)
pET28a-*tobR*	pET28a(+) derivative carrying *tobR*	This study

Table S2 Primers used in this study.

Primers	Sequences (5’→3’)
aprJ-F1	atgcgctccatcaagaagagcgatgcggtgaggcgacatgacg
aprJ-R1	ccacgtcgccggggatgggccgtggtcgt
aprJ-F2	gcccatccccggcgacgtggcggtggtact
aprJ-R2	tcctctagagtcgacctgcatccaccacgactgcgtggactc
aprK-F1	atgcgctccatcaagaagaggtgctcggcgaggtccacta
aprK-R1	aagtcgtctcgtgctggagtcggggaggtaa
aprK-F2	gactccagcacgagacgacttttggcgaagta
aprK-R2	tcctctagagtcgacctgcagctacgtgatcgtcctgctgaa
aprQ-F1	atgcgctccatcaagaagagccgggtcggtcacgtccatcg
aprQ-R1	gcggggcaggcacgccccagtggtcgtaggc
aprQ-F2	ctggggcgtgcctgccccgcgtggtgatggacta
aprQ-R2	tcctctagagtcgacctgcatggtctccggctcctcggg
aprI-F1	atgcgctccatcaagaagagcgggaagaacggtgatcgattc
aprI-R1	tcgttgggccacggtcgtcctccagtcgtc
aprI-F2	ggacgaccgtggcccaacgagtgaggactc
aprI-R2	tcctctagagtcgacctgcagtgtcagctcccacgaagactg
aprM-F1	atgcgctccatcaagaagagccgccggttcttcaccgagg
aprM-R1	ctcgtggccgcggtgcacgaggaactgggc
aprM-F2	tcgtgcaccgcggccacgagtccacgcagtc
aprM-R2	tcctctagagtcgacctgcatctaccggggccgcaaggtc
di-aprJ-F	cggccggttcgacgtgatct
di-aprJ-R	cacgtccaccacctgacgca
di-aprK-F	gacgtgatctacgcgaacctg
di-aprK-R	gttcgtcttcaccggcttcc
di-aprQ-F	gacccggacagttgggcctacg
di-aprQ-R	cggggctgcggatgctcgtgta
di-aprI-F	tgaccgtgatcagcaacgag
di-aprI-R	gatcagcgtcgagcagtacc
di-aprM-F	ctgctggcgagctgttccct
di-aprM-R	cctggagtacggccacaccg
28a-V-F	caaagcccgaaaggaagctgag
28a-V-R	gcgacccatttgctgtccac
28-tobR-F	gtggacagcaaatgggtcgcgtgcctcgtgacgggaaggt
28-tobR-R	cagcttcctttcgggctttgtcagtcctccccctcacgcc
ptobO-F	agtgctcccacaaccggattc
ptobO-R	aggcagatcgccagcaggac
v1139-F	tgcaggtcgactctagaggatc
v1139-R	ctcttcttgatggagcgcatg
tobR-F1	atgcgctccatcaagaagagggtcagcgaccggatccacg
tobR-R1	tccttcagcagccacctggggagcggttat
tobR-F2	cccaggtggctgctgaaggacgcgccgaac
tobR-R2	tcctctagagtcgacctgcaaacgccacggctacaccttcc

Primers	Sequences (5’→3’)
di-tobR-F	cagcgggtggtggacgttct
di-tobR-R	ccgccgcctgcaatgttcac
v152-F	atgtccgcctcctttggtcac
v152-R	tttgcgtattgggcgctctt
tobO-F1	atgcgctccatcaagaagagggtacggcgtgtcctccacg
tobO-R1	ccgcggacgctggctgcggcggttctactc
tobO-F2	accgccgcagccagcgtccgcggcgaaacc
tobO-R2	tcctctagagtcgacctgcaggaggcaccgttcggacacc
di-tobO-F	atctccgggaacgcgtggtg
di-tobO-R	tcctgcaacagggcgagcaa
ermE-tobR-F	tgaccaaaggaggcggacatgtgcctcgtgacgggaaggt
ermE-tobR-R	aagagcgcccaatacgcaaatcagtcctccccctcacgc
ptobE-F	gctactcccgccccttcgag
ptobE-R	gctgccgcagatcgagttgt
ptobT-F	cgtgaaggcgttgacgatcag
ptobT-R	gctcgtgcagcccgaagaac
ptobB-F	ctcggtcggcttctacctggc
ptobB-R	ggtgaggtcgaggtaggcgtt
ptobZ-F	ggttggccgtgttgtccgag
ptobZ-R	ttccgtgtgaaccgctcctc
ptobS1-F	gcacgaggactggtccaccc
ptobS1-R	ccgttgttgatcgcgaggtg
ptobM1-F	ggcggaccgaggacatcca
ptobM1-R	cgggacagccgcaggatct
ptobA-F	gcgtactggaagaccgcgac
ptobA-R	tccggtgtggacgtggtcaa
ptobO-F	agtgctcccacaaccggattc
ptobO-R	aggcagatcgccagcaggac
ermE-tobO-F	tgaccaaaggaggcggacatggaccgacagtggaggaaat
ermE-tobO-R	aagagcgcccaatacgcaaactcaccacaggcctcggat
V-kasOp-tobO-F	atttcctccactgtcggtccaaactcccccagtcctgcac
tobO-F	ggaccgacagtggaggaaat
qp-gapA-F	gccaacgaggagaagtacga
qp-gapA-R	ggttctggtcctgcgtgta
qp-tobO-F	cctgtggttcctcagcaacta
qp-tobO-R	cgtggtggagaaggtgttgt
qp-tobB-F	ccgaccaggcgttgttcta
qp-tobB-R	cgtggaactccgtggcata
qp-tobE-F	tggcacggcttcagttacc
qp-tobE-R	tcctggaggttctcgcagag
qp-tobM1-F	ctggctgaccgacctcttc
qp-tobM1-R	ctattcctgagggcggagac
qp-tobS1-F	cgcatggaactggtggaga

Primers	Sequences (5’→3’)
qp-tobS1-R	gaggagggtgttgagcagtt
qp-tobT-F	tgagccgtccacagagga
qp-tobT-R	tgggcaacaggatgtccag
qp-tobZ-F	agcggttcacacggaagaa
qp-tobZ-R	tgggcaacaggatgtccag

Table S3: Analysis of the gene products of the tobramycin gene cluster of *Streptoalloteichus tenebrarius* Tb. The table presents the results of BlastP analysis (Altschul et al. 1990).

Gene	Protein	Length (bp)	Predicted function	BlastP-Hits (Accession, percent identity)
tobE	TobE	1020	2-deoxy-scyllo-inosamine dehydrogenase	WP_253672087.1 (99.71)
tobT	TobT	1311	MFS transporter	WP_253672088.1 (99.77)
tobB	TobB	1188	glutamate-1-semialdehyde 2, 1-aminomutase	WP_253672089.1 (100%)
tobQ	TobQ	1527	paromamine 6'-oxidase	WP_253672090.1 (100%)
tobZ	TobZ	1713	carbamoyltransferase	WP_253672091.1 (100%)
tobS1	TobS1	1381	DegT/DnrJ/EryC1/StrS family aminotransferase	WP_253672092.1 (100%)
tobC	TobC	1161	2-deoxy-scyllo-inosose synthase	WP_253672093.1 (100%)
tobD2	TobD2	1044	Gfo/Idh/MocA family oxidoreductase	WP_253672094.1 (100%)
tobM2	TobM2	1263	putative 6-glucosyltransferase	WP_253672095.1 (100%)
tobN	TobN	786	PIG-L family deacetylase	WP_253672096.1 (100%)
tobS2	TobS2	1251	DegT/DnrJ/EryC1/StrS family aminotransferase	WP_253672097.1 (99.74%)
orf16	Orf16	372	Unknown	WP_253672098.1 (100%)
tobM1	TobM1	1266	glycosyltransferase involved in cell wall bisynthesis	MCP2261261.1 (100%)
tobL	TobL	1233	ATP-grasp domain-containing protein	WP_253672100.1 (99.76%)
tobU	TobU	981	DMT family transporter	WP_253672101.1 (100%)
tobA	TobA	1119	PLP-dependent aminotransferase	WP_253672102.1 (100%)
tobD3	TobD3	1077	alpha-hydroxy-acid oxidizing protein	WP_253672103.1 (100%)
tobO	TobO	984	TauD/TfdA family dioxygenase	WP_253672104.1 (100%)
tobR	TobR	492	Lrp/AsnC family transcriptional regulator	WP_253672105.1 (100%)

References

Altschul SF, Gish W, Miller W, Myers EW, Lipman DJ (1990) Basic Local Alignment Search Tool. J Mol Biol 215(3):403-410 doi:DOI 10.1006/jmbi.1990.9999

Sun JH, Kelemen GH, Fernández-Abalos JM, Bibb MJ (1999) Green fluorescent protein as a reporter for spatial and temporal gene expression in

A3(2). Microbiol-Uk 145:2221-2227 doi:Doi 10.1099/00221287-145-9-2221
